# New Cytotoxic Cerebrosides from the Red Sea Cucumber *Holothuria spinifera* Supported by *In-Silico* Studies

**DOI:** 10.3390/md18080405

**Published:** 2020-08-01

**Authors:** Reda F. A. Abdelhameed, Enas E. Eltamany, Dina M. Hal, Amany K. Ibrahim, Asmaa M. AboulMagd, Tarfah Al-Warhi, Khayrya A. Youssif, Adel M. Abd El-kader, Hashim A. Hassanean, Shaimaa Fayez, Gerhard Bringmann, Safwat A. Ahmed, Usama Ramadan Abdelmohsen

**Affiliations:** 1 Department of Pharmacognosy, Faculty of Pharmacy, Suez Canal University, Ismailia 41522, Egypt; reda.abdelhameed@pharm.suez.edu.eg (R.F.A.A.); enastamany@gmail.com (E.E.E.); dina_hal@pharm.suez.edu.eg (D.M.H.); am_kamal66@yahoo.com (A.K.I.); hasanean2000@yahoo.com (H.A.H.); 2 Department of Pharmaceutical Chemistry, Faculty of Pharmacy, Nahda University, Beni Suef 62513, Egypt; asmaa.aboulmaged@nub.edu.eg; 3 Department of Chemistry, College of Science, Princess Nourah bint Abdulrahman University, Riyadh 13414, Saudi Arabia; tarfah-w@hotmail.com; 4 Department of Pharmacognosy, Faculty of Pharmacy, Modern University for Technology and Information, Cairo 11566, Egypt; khayrya.youssif@gmail.com; 5 Department of Pharmacognosy, Faculty of Pharmacy, Deraya University, New Minia 61111, Egypt; ad_cognosy@yahoo.com (A.M.A.E.-k.); usama.ramadan@mu.edu.eg (U.R.A.); 6 Department of Pharmacognosy, Faculty of Pharmacy, Al-Azhar University, Assiut 71524, Egypt; 7 Institute of Organic Chemistry, University of Würzburg, Am Hubland, 97074 Würzburg, Germany; shaimaa.seaf@uni-wuerzburg.de; 8 Department of Pharmacognosy, Faculty of Pharmacy, Ain-Shams University, Cairo 11566, Egypt; 9 Department of Pharmacognosy, Faculty of Pharmacy, Minia University, Minia 61519, Egypt

**Keywords:** LC-HRESIMS, *Holothuria spinifera*, cerebrosides, molecular docking, cytotoxicity

## Abstract

Bioactivity-guided fractionation of a methanolic extract of the Red Sea cucumber *Holothuria spinifera* and LC-HRESIMS-assisted dereplication resulted in the isolation of four compounds, three new cerebrosides, spiniferosides A (**1**), B (**2**), and C (**3**), and cholesterol sulfate (**4**). The chemical structures of the isolated compounds were established on the basis of their 1D NMR and HRMS spectral data. Metabolic profiling of the *H. spinifera* extract indicated the presence of diverse secondary metabolites, mostly hydroxy fatty acids, diterpenes, triterpenes, and cerebrosides. The isolated compounds were tested for their in vitro cytotoxicities against the breast adenocarcinoma MCF-7 cell line. Compounds **1**, **2**, **3**, and **4** displayed promising cytotoxic activities against MCF-7 cells, with IC_50_ values of 13.83, 8.13, 8.27, and 35.56 µM, respectively, compared to that of the standard drug doxorubicin (IC_50_ 8.64 µM). Additionally, docking studies were performed for compounds **1**, **2**, **3**, and **4** to elucidate their binding interactions with the active site of the SET protein, an inhibitor of protein phosphatase 2A (PP2A), which could explain their cytotoxic activity. This study highlights the important role of these metabolites in the defense mechanism of the sea cucumber against fouling organisms and the potential uses of these active molecules in the design of new anticancer agents.

## 1. Introduction

Natural products have been a prime source of compounds with substantial structural diversity and numerous therapeutic activities [1], including plants, marine organisms, animals, and minerals [2]. Marine organisms can be considered as a great reservoir of new bioactive compounds that can aid in the prevention and treatment of different ailments, including cancer [3]. Research on marine-derived natural products emerged as early as the 19th century, and since 1980, the study of marine-derived natural products has led to the discovery of potential applications in drug development [4,5]. As a result of this intense research, more than 10,000 bioactive metabolites have so far been discovered [6]. The marine ecosystem is recognized as a treasure trove of unprecedented bioactive natural products and nutraceuticals with exceptional structural and chemical properties [7,8]. Marine organisms are commonly soft-bodied, and thus, tend to produce poisonous secondary metabolites or acquire them from microorganisms to protect themselves from predators [8]. Today, there is rising interest in exploiting marine diversity and complexity for drug discovery [9]. Many bioactive compounds have been isolated from different groups of marine organisms; among them are corals, tunicates, sponges, and sea cucumbers [6]. The Egyptian Red Sea coast and the Gulf of Aqaba host numerous marine communities, and one of the most abundant marine taxa in that area are the holothurians (sea cucumbers) [10]. They belong to the phylum Echinodermata, class Holothuroidea, and are commonly known as holothurians, comprising about 1200 known species worldwide [6]. Sea cucumbers constitute a huge group of organisms, from which a vast range of secondary metabolites have been isolated—among them, cerebrosides [11,12]. These compounds possess several pharmacologically relevant bioactivities, such as antiviral, antibacterial, antifungal, or anticancer effects [6]. It has also been reported that sea cucumber extracts show multiple biological activities, such as wound healing, antioxidant, and immunomodulatory effects [6]. Metabolomics encompass comprehensive analysis of the total number of metabolites within a biological sample using a specific set of parameters; metabolomics could serve as a reliable method to provide a picture of the central information of a cell, tissue, or whole organism [13]. In this paper, we report on research including bioactivity-guided fractionation complemented by LC-HRESIMS-assisted chemical investigation of the Red Sea cucumber *Holothuria spinifera,* leading to the isolation and structural characterization of three new cerebrosides, named spiniferoside A (1)—which is a mixture of three cerebrosides A1 (1a), A2 (1b), and A3 (1c)—spiniferoside B (2), and spiniferoside C (3), along with a fourth, a known sterol, cholesterol sulfate (4). The SET [Su(var)3-9, enhancer-of-zeste and Trithorax] is a protein domain that plays a key role in the methylation of histones, hence regulating gene expression. It is also a potent inhibitor of protein phosphatase 2A (PP2A), a serine/threonine enzyme with a tumor suppressing effect. In breast cancer, the SET protein was found to be overexpressed, and its knockdown decreased tumorigenesis. SET inhibits PP2A activity through binding to both N-terminus and C-terminus regions of PP2A. Given that PP2A maintains the activation of some oncogenic survival signals, SET is therefore an attractive and powerful therapeutic target for breast cancer therapy. In the same context, a recent topic that has gained interest in breast cancer research is the important role of the ceramide metabolism in this disease. Sphingolipid ceramide has long been described to activate PP2A through direct binding to SET oncoprotein [14,15].Our research efforts were oriented to discover bioactive drug candidates from the Red Sea marine organism, and supported by in silico studies, we have investigated a promising lead offered by sea cucumber *Holothuria spinifera* from the Red Sea, and three new cerebrosides were isolated. In vitro studies on the breast carcinoma cell line MCF-7 revealed that the new compounds displayed promisingantitumor activity comparable to the control drug doxorubicin. In the same context, and, to better understand the reasons for this cytotoxicity and the possible underlying mechanisms, investigations using molecular modeling research tools were carried out on the SET oncoprotein.

## 2. Results and Discussion

### 2.1. Structure Elucidation of the Isolated Compounds

**Figure 1:**
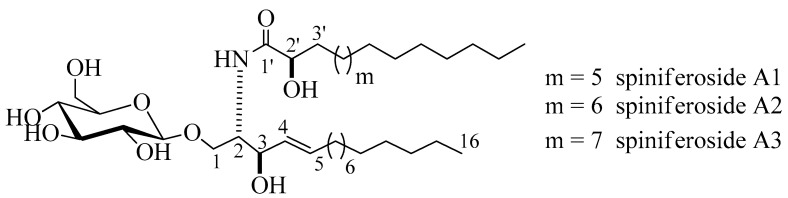
Chemical structures of spiniferoside A1–A3.

Compound **1** (Figure 1) was isolated as an amorphous white substance. It was identified as a cerebroside mixture **1** of three similar metabolites, compounds, **1a**, **1b**, and **1c**, all with a sphingosine-type moiety having the molecular formulae C_37_H_72_NO_9_, C_38_H_74_NO_9_, and C_39_H_76_NO_9_, respectively; two degrees of unsaturation in all three cases. The LC-HRESIMS displayed three peaks at *m/z* 674.5207, 688.5369, and 702.5513 [M + H]^+^ (Figure S1). The ^1^H and ^13^C NMR spectral data of compound **1** are listed in Table 1 (Figures S2–S5). The ^1^H NMR spectrum (measured in C_5_D_5_N, 400 MHz) of **1** indicated a sphingolipid skeleton, by the presence of an exchangeable proton signal of NH group at *δ*_H_ 8.37 (1H, d, *J* = 8.0 Hz) along with the typical aliphatic chain resonances, overlapping methyls at *δ*_H_ 0.85, and the long methylene chain protons at *δ*_H_ 1.25. Further characteristic NMR resonances were observed at *δ*_H_ 4.82 (1H, m, H-2), 4.82 (2H, m, H-3), 4.79 (2H, m, H-2’), 5.48 (1H, m, H-4), and 5.96 (1H, m, H-5), besides the signal for an anomeric proton at *δ*_H_ 4.53 (1H, d, *J* = 8.0 Hz, H-1’’), indicative of a sphingosine-type cerebroside. The ^13^C NMR spectrum (C_5_D_5_N, 100 MHz) showed signals at *δ*_C_ 14.1 for the methyl groups and at *δ*_C_ 173.4 for an amide carbonyl function. The resonances of the 2-amino-1,3,2′-triol part of the hydrocarbon chain were observed at *δ*_C_ 54.9 (C-2), 70.5 (C-1), 72.6 (C-3), and 72.6 (C-2’). The two methine groups resonated at *δ*_C_ 132.2 and 132.6 for (C-4) and (C-5), respectively. The position of the double bond was assigned at C-4 based on the typical biosynthetic pathway of ceramides as in [16]. The ^13^C NMR spectrum displayed characteristic signal corresponding to an anomeric carbon at *δ*_C_ 105.9 in addition to related downfield-shifted resonances at *δ*_C_ 75.2, 78.5, 71.5, 78.5, and 62.6, revealing the presence of a sugar moiety. The glucopyranosyl moiety was assigned to be *β*-configured based on the observed coupling constant of the anomeric proton at *δ*_H_ 4.53 (1H, d, *J* = 8.0 Hz, H-1’’) characterizing the di-axial interactions between H-1′’ and H-2′ and the chemical shift of the anomeric carbon *δ*_C_ 105.9 ppm (in the case of an *α*-configuration, the coupling constant should be 3.7 Hz, and the ^13^C NMR signal of the anomeric carbon should resonate at *δ*_C_ 98.5 ppm) [17,18]. In order to determine the length of the fatty acid chain, compound **1** was subjected to methanolysis followed by peak detection by LC-HRESIMS according to the method adopted by Sun and coworkers [19]. In brief, compound **1** was treated with aqueous HCl/MeOH for methanolysis, by which hydroxy acid methyl esters, sphingosine, and a sugar moiety were produced. LC-HRESIMS analysis of the fatty acid methyl esters of compound **1** molecular species mixture gave three molecular-ion peaks at *m/z* 273.2461, 287.2573, and 301.2729 [M]^+^, corresponding to the molecular formulas C_16_H_32_O_3_, C_17_H_34_O_3_, and C_18_H_36_O_3_ of the fatty acid methyl esters, methyl-2-hydroxypentadecanoate, methyl-2-hydroxyhexadecanoate, and methyl-2-hydroxyheptadecanoate, respectively. The relative configuration of the cerebroside moieties was deduced to be (2*S*, 3*R*, 4*E*, 2’*R*), as evidenced from the optical rotation +17.20 (c =1.00, MeOH) and the aforementioned ^13^C NMR signals (C-1, C-2, C-3, C-4, C-5, and C-2’) and their corresponding protons in the ^1^H NMR (measured in C_5_D_5_N) spectrum, which were in accordance with those of the known, previously reported sphingosine-type cerebrosides HLG-2 [20] and HPC-2 [12] possessing the same 2*S*,3*R*,4*E*,2’*R*-configuration. The sugar produced during the hydrolysis exhibited a comparable specific rotation +17.2 (c = 0.1%, H_2_O) as the standard, d-glucose. Moreover, the acetylated thiazolidine derivative of the obtained sugar was analyzed using a Cosmosil-5C_18_-AR-II column and it displayed the same t_R_ as the standard, d-glucose (19.7 min). From the aforementioned data, the structure of compound **1** was deduced to be a molecular species of the sphingosine-type glucocerebrosides possessing three different 2-hydroxy fatty acids with the molecular formulas C_37_H_72_NO_9_, C_38_H_74_NO_9_, and C_39_H_76_NO_9_. To the best of our knowledge, compound **1** (henceforth named spiniferoside A) is a new molecular species. Rooted in the considerable interest and importance of determining the molecular compositions of species of sphingolipids, the isolation and the structural elucidation of the cerebrosides in the molecular species **1** were conducted. Using a reversed phase HPLC adsorbent, **1** was separated into its three peaks and they were recovered to yield three cerebrosides spiniferoside, A1 (**1a**), spiniferoside A2 (**1b**), and spiniferoside A3 (**1c**) following the method described in [21]. Spiniferoside A2 was the major metabolite in the molecular species mixture with a molecular mass of m/z 688.5363 [M + H]^+^, while spiniferosides A1 and A3, with molecular masses of m/z 674.5207 [M + H]^+^ and 702.5513 [M + H]^+^ respectively, occurred in smaller quantities (Figures S9–S15).

**Figure 2:**
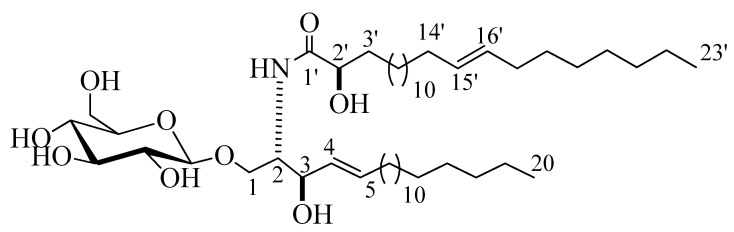
Chemical structure of the newly discovered compound **2,** spiniferoside B.

Compound **2** (Figure 2) was isolated as a white powder with a molecular formula of C_49_H_94_NO_9_, representing three degrees of unsaturation, as deduced from its ^1^H and ^13^C NMR spectral data and further confirmed by LC-HRESIMS analysis, which displayed a molecular ion peak at *m/z* 840.6923 [M + H]^+^ (Figure S16). The ^1^H and ^13^C NMR data of **2** were listed in Table 1 (Figures S17–S21). The ^1^H NMR spectrum (C_5_D_5_N, 400 MHz) displayed resonances typical of long methylene chain protons at *δ*_H_ 1.25, overlapped signals of methyl groups at *δ*_H_ 0.85, and a peak for an amide proton at *δ*_H_ 8.37 (1H, d, *J* = 8.0 Hz) indicative of a sphingolipid skeleton. Another signal characteristic of a sphingosine-type cerebroside appeared at *δ*_H_ 4.79 (1H, m, H-2), 4.79 (2H, m, H-3), 4.56 (2H, m, H-2’), and 5.96 (2H, m, H-4 and H-5), and the anomeric proton resonated at *δ*_H_ 4.98 (1H, d, *J* = 8.0 Hz, H-1’’). The ^13^C NMR spectrum in (C_5_D_5_N, 100 MHz) displayed an amide carbonyl at *δ*_C_ 175.7, two terminal methyl groups at δ_C_ 14.1, and characteristic resonances at *δ*_C_ 54.6 (C-2), 70.0 (C-1), 72.4 (C-2’), 72.5 (C-3), 131.9 (C-4), and 131.1 (C-5). The ^13^C NMR spectrum displayed the characteristic signal of an anomeric carbon *δ*_C_ 105.6, together with other downfield-shifted signals at *δ*_C_ 75.0, 78.4, 71.4, 78.5, and 62.5 indicating the presence of a sugar part. The observed coupling constant of the anomeric proton at *δ*_H_ 4.98 (1H, d, *J* = 8.0 Hz, H-1’’) characterizing the di-axial interaction between H-1′’ and H-2′, together with the chemical shift for the anomeric carbon *δ*_C_ 105.6, confirmed the *β*-configuration of the glucopyranosyl moiety (*α* glucopyranoside: *J* = 3.7 Hz; *δ*_C_ 98.5) [17,18]. The length of the fatty acid chain was determined by its methanolysis, followed by peak detection using LC-HRESIMS analysis for the obtained methyl ester of the fatty acid portion of **2**. As described in [19], compound **2** reacted with 5% methanolic HCl to produce the respective hydroxy acid methyl ester, sphingosine, and a sugar. LC-HRESIMS analysis of the fatty acid methyl ester of **2** exhibited a molecular ion peak at *m/z* 383.3547 [M]^+^ (Figure S25), corresponding to a C_24_H_46_O_3_ fatty acid methyl ester, (E)-methyl 2-hydroxytricos-15-enoate. Further confirmation of the double bond in the *α*-hydroxy fatty acid methyl ester was provided by GC–MS analysis. The *α*-hydroxy fatty acid methyl ester resulting after hydrolysis C_24_H_46_O_3_ with *m/z* 383.3547 [M]^+^ was subjected to oxidation by KmnO_4_, as described in [19], to yield fatty acid methyl ester C_23_H_44_O_2_ with *m/z* 352 that was subjected to GC–MS analysis; resulting fragments confirmed the position of the double bond by the presence of mass fragments at *m/z* 267 corresponding to a [C_17_H_31_O_2_^•^] fragment, *m/z* 253 corresponding to a [C_16_H_29_O_2_^•^] fragment, and *m/z* 125 corresponding to a [C_9_H_17_^•^] fragment (Figure S26). Therefore, the position of the double bond was authenticated in 15’ at (E) methyl 2-hydroxytricos-15-enoate. The sugar obtained from the hydrolysis displayed a specific rotation of +17.2 (c = 0.1%, H_2_O), which was identical to that of the standard, d-glucose. Furthermore, the t_R_ value of the acetylated thiazolidine derivative of the liberated sugar was similar to that of the standard, acetylated d-glucose (19.7 min). Like for compound **1**, the relative stereochemical details in the cerebroside moieties were determined to be (2*S*,3*R*,4*E*,2’*R*), by comparing its optical rotation and ^1^H NMR and ^13^C NMR spectral data (measured in C_5_D_5_N) with the correlatives reported previously. The optical rotation of +17.20 (c 1.00, MeOH) and the chemical shifts of C-1 (*δ*_C_ 70.0), C-2 (*δ*_C_ 54.6), C-3 (*δ*_C_ 72.5), C-4 (*δ*_C_ 131.9), C-5 (*δ*_C_ 131.1), and C-2’ (*δ*_C_ 72.4), along with the chemical shifts of their corresponding protons were similar to those of the known reported sphingosine-type glucocerebrosides HLG-2 [20] and HPC-2 [12], which possess 2*S*,3*R*,4*E*,2’*R*-configuration. From the above data, the structure of compound **2** was assigned as a cerebroside and it was given the name spiniferoside B, which is, to the best of our knowledge, a new compound.

**Figure 3:**
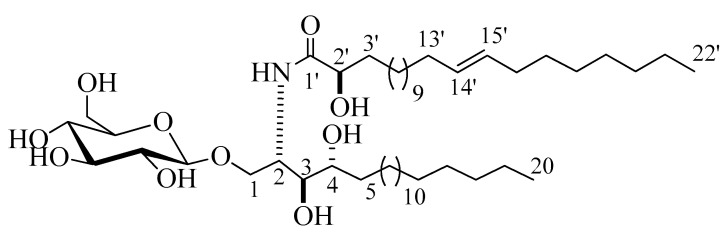
Chemical structure of compound **3**, spiniferoside C.

Compound **3** (Figure 3) was isolated as a white powder. Its molecular formula was deduced to be C_48_H_94_NO_10_, representing two degrees of unsaturation on the basis of its NMR analysis and further confirmed by the mass spectra with the appearance of a molecular-ion peak at *m/z* 844.6872 [M + H]^+^ (Figure S27). The ^1^H and ^13^C NMR spectral data of compound **3** are listed in Table 1 (Figures S28–S30). The ^1^H NMR spectrum (C_5_D_5_N, 400 MHz) of **3** displayed resonances typical of long methylene chain protons at *δ*_H_ 1.26, overlapped peaks of methyl groups at *δ*_H_ 0.87, and an exchangeable proton of an NH group at *δ*_H_ 8.60 (1H, d, *J* = 8.0 Hz), indicating the presence of a sphingolipid skeleton. In addition, several multiplets at *δ*_H_ 5.30 (1H, br,m, H-2), 4.20 (1H, m, H-4), 4.35 (1H, m, H-3), 4.34 (1H, m, H-1), and 4.74 (1H, t, *J* = 8.0, H-2’) were observed, along with an anomeric proton at *δ*_H_ 4.97 (1H, d, *J* = 8.0 Hz, H-1’’), exhibiting a phytosphingosine-type cerebroside. The ^13^C NMR spectrum (measured in C_5_D_5_N, at 100 MHz), displayed an amide carbonyl resonating at *δ*_C_ 175.7, two terminal methyl groups at *δ*_C_ 14.1, and the resonances characteristic of the 2-amino-1,3,4,2′-tetrol part of the hydrocarbon chain at *δ*_C_ 51.4 (C-2), 70.2 (C-1), 72.1 (C-4), 72.2 (C-2’), and 74.9 (C-3). The ^13^C NMR spectrum displayed the characteristic signal of the anomeric carbon at *δ*_C_ 105.1, together with downfield-shifted signals at *δ*_C_ 75.0, 78.4, 71.4, 78.5, and 62.5 of the carbon skeleton of the sugar part. The chemical shift of the anomeric carbon at *δ*_C_ 105.1, along with the coupling constant of the anomeric proton at *δ*_H_ 4.97 (1H, d, *J* = 8.0 Hz, H-1’’) characterizing the di-axial interaction between H-1′’ and H-2′, confirmed the *β*-configuration of the glucopyranosyl moiety (for an *α*-glucopyranoside: *J* = 3.7 Hz; *δ*_C_ 98.5 ppm) [17,18]. As for compound **2**, the length of the fatty acid chain was determined by its methanolysis, while ensuing peak detection using LC-HRESIMS analysis for the fatty acid methyl ester obtained from **3**. According to the method described in [19], compound **3** reacted with 5% methanolic HCl, giving the corresponding hydroxy acid methyl ester, phytosphingosine, and a sugar molecule. The LC-HRESIMS spectrum of the fatty acid methyl ester of **3** exhibited a peak with a molecular ion of m/z 369.3358 [M]^+^ (Figure S32) corresponding to a C_23_H_44_O_3_ fatty acid methyl ester, (E)-methyl 2-hydroxydocos-14-enoate. Further confirmation of the double bond in *α*-hydroxy fatty acid methyl ester was provided by GC–MS analysis. The *α*-hydroxy fatty acid methyl ester resulted after hydrolysis C_23_H_44_O_3_ with *m/z* 369.3358 [M]^+^ was subjected to oxidation by KmnO_4_, as described in [19], to yield fatty acid methyl ester C_22_H_42_O_2_ with *m/z* 338 that was subjected to GC–MS analysis; resulting fragments confirmed the position of the double bond by the presence of mass fragments at *m/z* 239 corresponding to a [C_15_H_27_O_2_^•^] fragment, *m/z* 139 corresponding to a [C_10_H_19_^•^] fragment, and *m/z* 125 corresponding to a [C_9_H_17_^•^] fragment (Figure S33). Therefore, the position of the double bond was authenticated in 14’ at (E) methyl 2-hydroxydocos-14-enoate. The sugar liberated from the hydrolysis displayed a specific rotation of +17.2 (c = 0.1%, H_2_O), which was identical to that of the standard, d-glucose. Furthermore, the t_R_ of the acetylated thiazolidine derivative of the liberated sugar was similar to that of the standard, d-glucose (19.7 min). The relative configurations at the stereocenters of the cerebroside moieties were assigned to be (2*S*, 3*S*, 4*R*, and 2’*R*), as evidenced by the comparison of their physical properties and NMR data (measured in C_5_D_5_N) with those of analogs reported in the literature [20]. The recorded optical rotation +17.40 (c 1.00, MeOH) and the aforementioned ^13^C NMR signals (C-1, C-2, C-3, C-4, and C-2’), along with the chemical shifts of their corresponding protons in the ^1^H NMR (measured in C_5_D_5_N) spectrum, were in line with those reported for cerebroside HLG-1 [20] and HPC-3 [12]. From the above data, the structure of compound **3** was assigned as presented in Figure 3. To the best of our knowledge, it was a new compound, henceforth named spiniferoside C.

**Figure 4:**
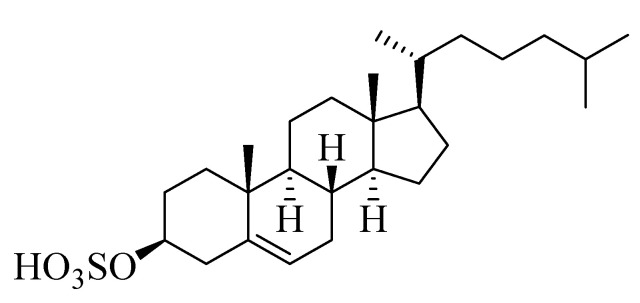
Chemical structure of compound **4**, cholesterol-3-O-sulfate.

Compound **4** (Figure 4) was isolated as a white powder. Derived from its NMR spectral data, the molecular formula of **4** was C_27_H_46_O_4_S, representing five double bond equivalents. The interpretation of the ^1^H NMR spectrum of compound **4** (Figure S34) indicated the presence of two methyl singlets at *δ*_H_ 0.62 and 0.75 assigned to CH_3_-19 and CH_3_-18, respectively, along with three methyl doublets at *δ*_H_ 0.85, 0.84, and 0.91 assigned to CH_3_-21, CH_3_-26, and CH_3_-27, respectively, which was in agreement with the presence of a steroidal nucleus [22]. Moreover, a trisubstituted olefinic proton at *δ*_H_ 5.28 (m) assigned to H-6 was observed in addition to an oxygenated methine at *δ*_H_ 3.93 (m), corresponding to an axial proton next to the O-sulfate group at C-3. The ^13^C NMR spectrum (Figure S35) indicated the presence of five methyl groups (CH_3_), eleven methylenes (CH_2_), eight methines (CH), and three quaternary carbons (C), besides an oxymethine carbon at *δ*_C_ 78.4 assigned to be C-3, to which the sulfate group was attached. Comparing the chemical shift values of H-3/C-3 with the data reported in [23] suggested the presence of a cholesterol sulfate structure. The afore-mentioned ^1^H NMR and ^13^C NMR spectral data came into line with those reported for cholesterol sulfate [23], so compound **4** was identified as cholesterol-3-O-sulfate.

### 2.2. Metabolic Profiling

The crude extract of *H. spinifera* was tested for its in vitro cytotoxicity on five cancerous cell lines: HepG2 (liver cancer cell line), MCF7 (breast cancer cell line), PC3 (prostate cancer cell line), HCT 116 (colon cancer cell line), and HeLa (cervix cancer cell line). This test was performed by applying the sulforhodamine B (SRB) assay using the method adapted by Skehan et al. [24], which follows the protocol described by Vichai and Kirtikara [25]. An ELISA reader was used to measure the color intensity, and the corresponding IC_50_ values (extract concentration which reduces survival of cancer cells to 50%) were calculated.

The crude extract of *H. spinifera* displayed potent cytotoxic activity on MCF7 with an IC_50_ value of 4.58 µg/mL and weak activity on HCT 116 with an IC_50_ of 19.4 µg/mL. On the contrary, the extract exhibited no significant cytotoxicity on PC3, HepG2, and HeLa cells, achieving IC_50_ values of 44.7, 24.5, and 32.3 µg/mL respectively, as established by the U.S. National Cancer Institute (NCI; the criterion of cytotoxicity is an IC_50_ < 20 µg/mL), in a preliminary evaluation [26]. Upon treatment with the crude extract of *H. spinifera*, the MCF-7 cells exhibited an enhanced rate of cell death with a lower concentration of the extract compared to that in other tested cell lines.

Based on the aforementioned results and motivated by the potent cytotoxicity of the *H. spinifera* extract on MCF-7 cells, we identified the secondary metabolites in the extract using the LC-HRESIMS technique.

LC-HRESIMS metabolic analysis is a new strategy for the comprehensive phytochemical characterization of complex crude extracts, along with targeting specific metabolites that can be associated with certain biological activities prior to sometimes tedious and time-consuming purification procedures. By using combinations of different analytical methods, the bioassay-guided isolation route is shortened and putative identification of potentially new natural products is rapidly achieved. Since the metabolic profile in each extract varies by its physical nature and ionization potential, both the positive and negative ionization modes were combined, so that detection of the maximum possible metabolites was accomplished. A portion of 57% of the metabolites were detected in positive mode and 39% of the metabolites were found in negative mode, while only 4% were detected in both modes. Prior to dereplication, molecular formula prediction was done utilizing the MZmine algorithm, which employs a combination of empirical techniques that include isotope pattern matching. Using the positive and negative mode electrospray ionization, with spectral data at a MW tolerance within 5 ppm, known compounds were tentatively identified. In the data processing step, two parameters were used, the “retention time” and the “*m/z* tolerance range.” If the variance was deemed to be too high (i.e., 20% for LC-HRESIMS data), the feature was removed from the analysis. This processing step was performed at the start of the data analysis process.

**Figure 5:**
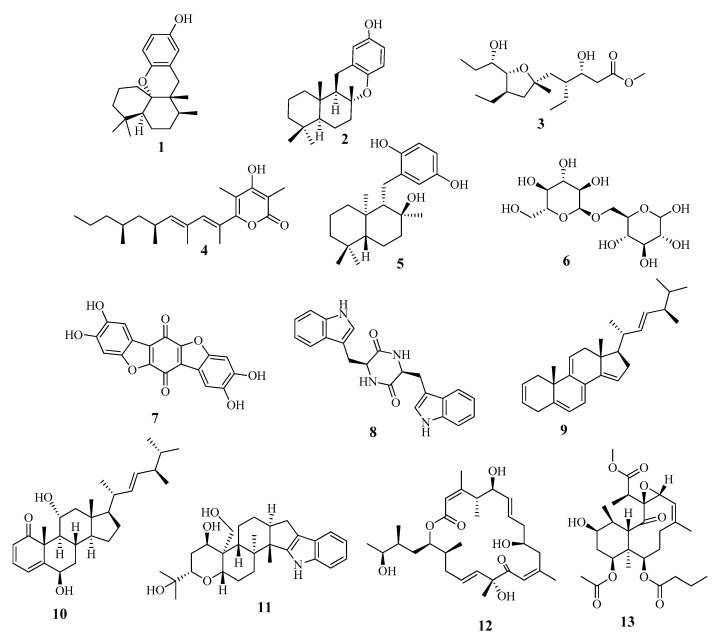
Dereplicated metabolites from *Holothuria spinifera*.

The metabolomic profiling of *Holothuria spinifera* using LC-HRESIMS led to the identification of secondary metabolites belonging to the following chemical groups: fatty acids, phenolic diterpenes, and triterpenes. The metabolites in the extract (Table 2 and Figure 5) were tentatively identified by searching in databases, e.g., in the Dictionary of Natural Products (DNP), and in (METLIN).

According to the literature, the majority of the identified secondary metabolites displayed cytotoxic and anticancer effects. For instance, Aureol in vitro cytotoxic activities were tested against human liver carcinoma (Hepa59T/VGH), human oral epidermoid carcinoma (KB), and human cervical epithelioid carcinoma (Hela) tumor cell lines and it showed promising cytotoxic activities. [27]. Assays on cytotoxic activity were performed for epichromazonarol against P-388, A-549, HT-29, and MEL-28 cells, and in all cases an IC_50_ = 15.9 μM was obtained for epichromazonarol [28], while Plakortether E was evaluated for cytotoxic activity against two different cell lines: WEHI 164 (murine fibrosarcoma) and RAW 264-7 (murine macrophage). Plakortether E proved to be selectively active against the second cell line [29]. Moreover, stoloniferone O, imposed significant antitumoral effects on HT-29 and P-388 cell lines [30]. Furthermore, the antiangiogenesis activity of thelephoric acid was reported, as it was evaluated using a tyrosine kinase assay kit with recombinant human VEGFR2. Since VEGFR2, a principal regulator of angiogenesis, is highly expressed in umbilical vein endothelial cells, thelephoric acid was examined on cell growth of HUVECs by a WST-8 assay. As a result, it exhibited antiproliferative activity in HUVECs. It also markedly inhibited cell invasion [31]. In addition, terpendole F inhibited cholesterol acyl-transferase (ACAT), a crucial enzyme in cancer progression [32].

All the above-mentioned data supported the displayed cytotoxicity of the *H. spinifera* extract.

### 2.3. In Vitro Evaluation of the Antitumor Activities of the Isolated Compounds

The cytotoxicity levels of ceramides and cerebrosides on wide array of cancer cell lines have previously been reported [42,43,44]. SJG-2 is one example of a ganglioside molecular species recently isolated from *Stichopus japonicas* possessing neurogenic activity against the rat pheochromocytoma cell line PC12 [45]. Jia and coworkers proved the effectiveness of a glucocerebroside mixture isolated from the sea cucumber *Cucumaria frondosa* on the HepG2 liver cancer cell line [46]. The ophidiacerebrosides, which are cerebroside molecular species isolated from the African starfish *Narcissia canariensis*, displayed promising cytotoxicity on multiple myeloma (KMS-11), glioblastoma (GBM), and colorectal adenocarcinoma (HCT-116) cell lines [47]. Therefore, compounds **2**, **3**, and **4** and the spiniferoside A molecular mixture **1** isolated from the sea cucumber *Holothuria spinifera*, were assessed for their cytotoxic effect on MCF-7 cancer cell line by applying the sulforhodamine B (SRB) assay using the method adapted by Skehan et al. [24], which follows the protocol described by Vichai and Kirtikara [25]. The MCF-7 cancer cells were chosen particularly for the cytotoxicity evaluation rather than any cancerous cell lines based on our results, which revealed the potent cytotoxicity of *H. spinifera* on MCF-7 cells. As presented in Table 3, all four compounds showed promising anticancer activities against cells of the MCF-7 breast cancer cell line. Compounds **2** and **3** demonstrated promising cytotoxic activities against MCF-7 cancer cells, with IC_50_ values of 8.13 and 8.27 µM respectively compared to the standard drug doxorubicin with IC50 values of 8.64 µM. Compound **1** showed a slightly lower effect, with an IC_50_ value of 13.83µM. The exhibited cytotoxicity of compound **1** could be attributed to the major component spiniferoside A***2***, as reported in the literature [47,48,49]. Cholesterol sulfate (**4**) exhibited the least cytotoxicity, with an IC_50_ value of 35.65 µM.

Ceramide acts as a cytotoxic agent and tumor-suppressor by induction of apoptosis. It was observed that treatment with exogenous ceramide activates a cascade of caspases, protein kinases, and phosphatases and leads to apoptosis [50]. On the other hand, glucosylceramide exerts its cytotoxic effect by arresting cell renewal capacity instead of induction of apoptosis. This could be explained in the light of the fact that sphingoid bases were reported to induce cell cycle arrest at G2/M phase. The presence of a glucose moiety increases the water-solubility of the compound, enhancing its membrane permeability. As a consequence, it is possible that a small amount of ceramide penetrates into cells, and then hydrolyzes to its sphingoid base, which in turn affects the cell physiology [51].

Doxorubicin was used as a standard drug in our cytotoxic assay based on the fact that it represents a pillar of various cancer treatment protocols, including breast cancer [52]. It is known that doxorubicin increases endogenous ceramide in drug-sensitive MCF-7, resulting in apoptosis [53] by induction of ceramide *de novo* synthesis [50].

Impairment of ceramide metabolism in cancer cells is an escape pathway necessary for their survival which leads to drug resistance. As a consequence, tumor progression and metastasis occur [54]. To overcome chemotherapy resistance, drug combinations were used. A recent study revealed that combining exogenous C6-ceramide sensitizes multiple cancer cell lines to doxorubicin or etoposide. In addition, combining sorafenib with nano liposomal ceramide enhances sensitivity to sorafenib in other cancer cell types, including breast carcinoma and melanoma [50].

Therefore, the isolated cerebrosides **2** and **3** could be used as an adjuvant therapy with doxorubicin to overcome drug resistance and increase the sensitivity to doxorubicin. In the future, a comparative pharmacological study will be conducted to evaluate the in vivo efficacy of the isolated cerebroside as an anticancer agent individually and in combination of doxorubicin to verify our assumption.

### 2.4. Molecular Docking Studies

A literature survey revealed that the SET oncoprotein plays a role in cancer progression by affecting multiple cellular processes through the inhibition of tumor suppressor protein phosphatase 2A (PP2A) and metastasis suppressor nm23-H1, or through alterations of inhibitory ceramide synthesis [55,56]. Sphingolipid ceramide has been reported to activate PP2A via direct interaction between them. Recently it has been revealed that an additional mechanism which involves the direct binding of ceramide with the SET protein may contribute to the activation of ceramide to PP2A [57]. Molecular docking studies suggested that the SET domain possesses a hydrophobic ceramide-binding pocket characteristic of the lipid binding pocket.

For this reason, targeting the inhibition of SET would have a significant and effective role in inhibiting the proliferation of cancer cells. The ability of the cerebrosides **1**, **2**, and **3**, and the sterol sulfate **4** to bind to the SET oncoprotein was examined by molecular docking. To further estimate a predictive picture concerning the site where the natural compounds are binding to the SET oncoprotein, docking scores for the natural compounds with the ligand are provided (Table 4).

**Figure 6:**
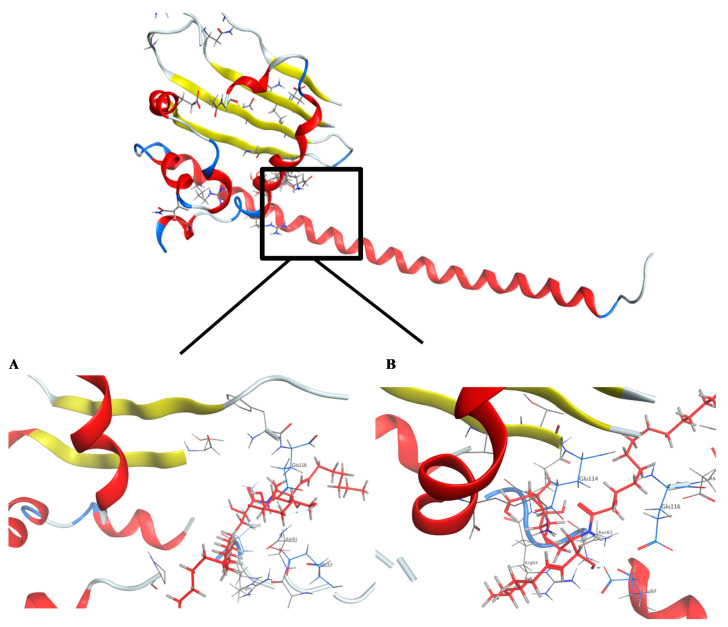
Crystal structure of the predicted docking pose (in blue) of (**A**) compound **2** and (**B**) compound **3** with the SET oncoprotein (PDB code: 2E50).

In this study, the docking pose of spiniferoside A (**1**) showed the hydrophobic skeleton, which was stabilized by the hydrophobic interactions with the amino acids Phe 68 and Pro 214. Per the predicated binding pose of spiniferoside C (**3**), two hydrogen bond donors were formed between the hydroxy group of **3** and the carbonyl oxygens of Asn 61 and Glu 57 with distances of 3.22 and 2.64 Å, respectively. Additionally, an extra hydrogen bond acceptor was found to be formed with the Glu 116 residue (Figure 6). This may explain the potent inhibitory activity of spiniferoside C (**3**) against the breast cancer cell line with an IC_50_ value of 8.27 µM. Spiniferoside B (**2**), on the other hand, formed two hydrogen bonds with Asn 61 and Glu 57 (Figure 6). These interactions may be missing, with relatively low binding energy scores, in cholesterol sulfate (**4**), which showed the least inhibitory activity against the MCF-7 cancer cell line. Based on molecular modeling, the compounds exhibited their anti-tumor activity through targeting the PP2A inhibitor, SET. Overexpression of the SET oncoprotein was reported in breast cancer cell lines, including MCF-7 [58,59]. The use of SET antagonists and PP2A activators was postulated as a relatively novel strategy for breast cancer therapy [59]. In a study that involved MDA-MB-231 breast adenocarcinoma cell line, targeting SET inhibited cellular proliferation and decreased cellular migration and invasion via activation of the tumor suppressors PP2A and nm23-H1 [55]. Suppression of SET reversed the resistance in MCF-7/PTX human breast carcinoma [60]. The pronounced cytotoxic effect of SET inhibitors in breast cancer might be explained by the concomitant activation of PP2A, a suggested therapeutic target in resistant MCF-7 cells [61]. Inhibition of PP2A has been associated with poor prognosis of breast cancer [62]. PP2A regulates the expression of estrogen receptor (ER), a key molecular determinant of breast cancer status and survival [62,63]. PP2A activation was raised as an effective treatment target in MCF-7 breast cancer cell line through its mediation of ERα expression via modulating ER mRNA stability [64].

## 3. Materials and Methods

### 3.1. General Experimental Procedures

^1^H NMR (400 MHz) and ^13^C NMR (100 MHz) spectra were recorded using the residual solvent signal as an internal standard on a Varian AS 400 (Varian Inc., Palo Alto, CA, USA). High-resolution mass spectra were recorded using a Bruker BioApex (Bruker Corporation). Pre-coated silica gel G-25 UV254 plates were used for thin-layer chromatography (TLC) (20 cm × 20 cm) (E. Merck, Darmstadt, Germany). Silica gel (Purasil 60Å, 230–400 mesh) was used for flash column chromatography (Whatman, Sanford, ME, USA). Semi-preparative high-performance liquid chromatography (HPLC) was performed on a Cosmosil 5 C18–MS–II (150 × 4.6 mm) at a flow rate of 0.5 mL/min. The column was equipped with a TOSOH RI-8020 detector and a JASCO BIP-I HPLC pump [20].

### 3.2. Sea Cucumber Material

The sea cucumber *Holothuria spinifera* was collected from Sharm El Sheikh in the Egyptian Red Sea. The material was air-dried and stored at −24 °C until further processing. Identification of the sea cucumber was performed by Dr. Tarek Temraz, Marine Science Department, Faculty of Science, Suez Canal University, Ismailia, Egypt. Voucher specimens were deposited at the Herbarium Section of the Pharmacognosy Department, Faculty of Pharmacy, Suez Canal University, Ismailia, Egypt under the registration number SAA-129.

### 3.3. Extraction and Isolation

Material of the sea cucumber *Holothuria spinifera* (2 kg) was frozen and chopped into small pieces, and then extracted with a mixture of MeOH/CH_2_Cl_2_ (1:1) (3 × 2 L) at room temperature. The resulting crude extract was evaporated under vacuum to afford 100 g of a dark-greenish residue. The extract was slurred with a small portion of silica gel. The mixture was transferred to the top of a sintered-glass Büchner funnel (15 cm × 10 cm) packed with 300 g of silica gel and connected to a vacuum pump. Fractionation was performed by step gradient elution using a non-polar solvent (*n*-hexane) with increasing the polarity using ethyl acetate (EtOAc) and then MeOH to give nine fractions: (HS-1–HS-9). Fraction HS-5 (75:25 EtOAc:MeOH) (2.52 g) was placed on a silica gel column and eluted initially with 100% CHCl_3_, followed by gradient systems of CHCl_3_:MeOH to (65:35), which yielded eight subfractions (HS-5-S-1 to HS-5-S-8). Subfraction HS-5-S-3 (150 mg) was chromatographed on silica gel using gradient systems of CHCl_3_:MeOH starting with (90:10) to (65:35) followed by a Sephadex LH-20 column for final purification using CHCl_3_:MeOH (1:1) isocratic elution to obtain compound **1** (17 mg, white amorphous powder). Compound **1**, as a molecular species mixture (3 mg) was finally resolved using semi-preparative HPLC (Cosmosil 5 C18, 100% MeOH) to afford spiniferoside A2 (**1b**, 0.9 mg), spiniferoside A1 (**1a**, 0.12 mg), and spiniferoside A3 (**1c**, 0.3 mg).

Subfraction HS-5-S-4 (200 mg) was re-chromatographed on a silica gel column using gradient systems of CHCl_3_:MeOH starting with (90:10) to (65:35) followed by a Sephadex LH-20 column twice using CHCl_3_:MeOH (1:1) isocratic elution to obtain compound **2** (15 mg, white amorphous powder). Subfraction HS-5-S-6 (250 mg) was subjected to silica gel column chromatography using gradient systems of CHCl_3_:MeOH starting with (90:10) to (65:35) followed by purification on Sephadex LH-20 twice using CHCl_3_:MeOH (1:1) isocratic elution to obtain compound **3** (19 mg, white amorphous powder). Fraction HS-4 (100% EtOAc) (0.83 g) was placed on a silica gel column and eluted initially with 100% CHCl_3_, and then with gradient systems of CHCl_3_:MeOH until (65:35); the effluent yielded eight subfractions (HS-4-H-1 to HS-4-H-8). Subfraction HS-4-H-6 (300 mg) was re-chromatographed on silica gel using gradient systems of CHCl_3_:MeOH starting with (90:10) to (65:35) and then subjected to a Sephadex LH-20 column chromatography for final purification using CHCl_3_:MeOH (1:1) isocratic elution to obtain compound **4** (40 mg, white amorphous powder).

### 3.4. Cerebroside Hydrolysis

Compounds **1**, **2**, and **3** were subjected to methanolysis, in which 2 mg each of the respective compounds was heated with 5% HCl in MeOH (0.5 mL) at 70 °C for 8 h in a sealed small-volume vial. The reaction mixture was extracted with *n*-hexane, and the hexane layer was evaporated under vacuum until dryness to give a mixture of FAM (fatty acid methyl esters) for LC-HRESIMS analysis. For further confirmation of the double bond in fatty acid side chain in compounds **2** and **3**, *α*-hydroxy fatty acid methyl esters obtained from hydrolysis were subjected to Lemieux oxidation. Hence, 0.023 mol/L aqueous KMnO_4_ and 0.09 mol/L NaIO_4_ (2.0 mL) were slowly added to the *α*-hydroxy fatty acids/methyl esters mixture, *t*-BuOH (1.0 mL), and 0.04 mol/L aqueous K_2_CO_3_ (0.5 mL). Then, the mixture was stirred for 18 h at 37 °C, quenched with 2.5 mol/L H_2_SO_4_ (0.1–0.3 mL) and saturated aqueous Na_2_SO_3_, and then extracted with Et_2_O. The organic layer was dried over Na_2_SO_4_. Finally, the concentrated, dried residue was esterified with excess CH_2_N_2_ in Et_2_O overnight. The resulting fatty acid methyl esters were used for GC–MS analysis [19]. The identification of the fatty acids and methyl esters was de-convoluted using AMDIS software (www.amdis.net) and identification was done by retention indices (mass spectrum matching to authentic standards; Wiley spectral library collection and NSIT library database).

### 3.5. Identification of the Sugar Moiety in Compounds 1, 2, and 3

In a sealed small-volume vial, 5 mg of each compound **1**, **2**, and **3** was heated in 5% HCl/MeOH (0.5 mL) at 70 °C for 8 h. CHCl_3_ was used to extract the reaction mixture for the removal of the fatty acids released. The methanolic layer was then neutralized with Ag_2_CO_3_ to provide the methylated sugar followed by HPLC checking (Cosmosil-sugar-D, 4.6 ID X 250 mm, 1 mL/min, RI detector, 95% acetonitrile) against the standards, glucose and galactose. Glucose displayed a retention time of 4.62 min, which was found as being our sample retention time; meanwhile, the retention time of galactose was 4.76 min.

### 3.6. Determination of the Configuration of the Sugar Moiety in 1, 2, and 3

For compounds **1**, **2**, or **3, 2** mg each was hydrolyzed by heating in 0.5 M HCl (0.1 mL) and then neutralized with Amberlite IRA400. After drying in vacuo, the residue was dissolved in pyridine (0.1 mL) containing L-cysteine methyl ester hydrochloride (0.5 mg) and heated at 60 °C for 1 h. A 0.1 mL solution of *o*-tolylisothiocyanate (0.5 mg) in pyridine was added to the mixture, which was heated to 60 °C for 1 h. The reaction mixture was directly analyzed by reversed-phase HPLC (Cosmosil 5C_18_-AR-II, 4.6 ID X 250 mm, 0.8 mL/min, λ = 250 nm, 25% acetonitrile in 50 mM H_3_PO_4_). The same derivatization procedures were done for 5 mg of standards, d-glucose (t_R_ = 19.7 min), l-glucose (t_R_ = 19.5 min), d-galactose (t_R_ = 18.6 min), and l-galactose (t_R_ = 19.3 min), evidencing the sugar portion to be d-glucose in all three cases.

### 3.7. Metabolomic Profiling

Metabolic profiling of the sea cucumber material was performed using the method described by Elsayed et al. [65]. These files were imported for peak picking, deconvolution, deisotoping, alignment, and formula prediction into the data mining program MZmine 2.10. Comparison with the MarinLit database and the Dictionary of Natural Products (DNP) 2015 achieved dereplication of a broad array of compounds.

### 3.8. Spectroscopic Data

Compounds 1, 2 and 3; were isolated as white powder; their ^1^H NMR (C_5_D_5_N, 400 MHz) and ^13^C NMR (C_5_D_5_N, 100 MHz) spectral data—see Table 1.

Compound 4; was isolated as white powder; ^1^H NMR (DMSO, 400 MHz) and ^13^C NMR (DMSO, 100 MHz) spectral data were similar to those reported in the literature [23].

^1^H NMR (DMSO, 400 MHz); δ_H_ 0.91–1.89* (2H, H-1), 1.23–1.97*(2H, H-2), 3.93 (1H, m, H-3), 2.35–2.38* (2H, H-4), 5.28 (1H, m, H-6), 1.94–2.33* (2H, H-7), 1.15–2.33* (1H, H-8), 1.15–2.33* (1H, H-9), 0.91–1.07* (2H, H-11), 1.15–2.38* (2H, H-12), 1.15–2.38* (1H, H-14), 1.15–2.38* (2H, H-15), 1.15–2.38* (2H, H-16), 1.15–2.38* (1H, H-17), 0.75 (3H, s, H-18), 0.99 (3H, s, H-19), 1.15–2.38* (1H, H-20), 0.85* (3H, H-21), 1.15–2.38* (2H, H-22), 1.15–2.38* (2H, H-23), 1.15–2.38* (2H, H-24), 1.15–2.38* (1H, H-25), 0.84–0.88* (3H, H-26), 0.84–0.88* (3H,H-27).

* The overlapped signals are reported without multiplicity

^13^C NMR (DMSO, 100 MHz); δ_C_ 38.2 (C-1), 31.9 (C-2), 78.4 (C-3), 42.1 (C-4), 143.8 (C-5), 124.2 (C-6), 40.0 (C-7), 34.5 (C-8), 52.7 (C-9), 38.2 (C-10), 22.1 (C-11), 25.5 (C-12), 38.4 (C-13), 56.8 (C-14), 25.5 (C-15), 21.7 (C-16), 56.8 (C-17), 14.8 (C-18), 21.7 (C-19), 34.8 (C-20), 21.7 (C-21), 34.5 (C-22), 23.9 (C-23), 40.0 (C-24), 27.0 (C-25), 22.9 (C-26), 22.9 (C-27).

### 3.9. Cytotoxicity Assays

The cyctotoxic activities of *H. spinifra* MeOH/CH_2_Cl_2_ (1:1) crude extract and the isolated compounds **1**, **2**, **3**, and **4** were investigated by the sulforhodamine B (SRB) assay, as mentioned in Skehan et al. [24], according to the method described by Vichai and Kirtikara [25]. The cytotoxicity of the crude extract was tested on HepG2 (liver cancer cell line), MCF7 (breast cancer cell line), PC3 (prostate cancer cell line), HCT 116 (colon cancer cell line), and HeLa (cervix cancer cell line). For the evaluation of the cytotoxicity of the isolated compounds, MCF-7 cancer cells were chosen; 96-well microtiter plates were used to seed cells at an initial concentration of 3 × 10^3^ cell/well in 150 µL of fresh medium and left for 24 h to get attached to the plates. The drugs were applied to the plates at serial different concentrations of 0, 5, 12.5, 25, and 50 µg/mL, and then all plates were incubated for 48 h. The cells were fixed with 50 μL of cold trichloroacetic acid 10% for 1 h at 4 °C, and then distilled water was used to wash the plates (automatic washer Tecan, Germany). SRB (50 μL, 0.4%) dissolved in 1% acetic acid used to stain the plates for 30 min at room temperature; then the plates were washed with 1% acetic acid and air-dried; 100 μL/well of 10 M tris base (pH 10.5) was used to solubilize the dye, and the optical density (OD) of each well was measured spectrophotometrically at 570 nm using an ELISA microplate reader (Sunrise Tecan reader, Germany). The mean background absorbance was automatically taken and each drug’s mean value of concentration was calculated. The experiment was done three times, and then the IC_50_ values were calculated.

### 3.10. Molecular Docking Studies

The crystallographic structure of the SET protein, inhibitor of PP2A, in complex with its ligand, was available from the Protein DataBank [66]. The structure arrangement process was used to revise the protein errors in addition to the creation of a reasonable protein structure that was set up on default rules on MOE (Molecular Operating Environment). Removal of water molecules and the ligands that were not involved in the binding was carried out using quick preparation protocol for the preparation of the protein. Finally, the Gasteiger methodology was applied to calculate the partial charges of the protein. Molecular docking studies were performed using Molecular Operating Environment 2019.0101 software [67]. The ligand coordinates of compounds **1b**, **1b**, **1c**, **2**, **3**, and **4** were built using ChemDraw Ultra 11.0. Their protonation, the atom correction, and bond types were then defined; hydrogen atoms were added; protonation, and final minimization were performed (AMBER10, gradient: 0.01). The docking experiment on the SET gene protein was performed by redocking the ligand on in the PDB file 2E50, after which the ligand was deleted. The default Triangle Matcher placement method was selected for docking. GBVI/WSA dG scoring function, which determines the free energy of binding of the ligand from a given pose, was chosen to rank the final poses. The ligand complex with the protein having the lowest S-score was selected. The redocking of the ligand with its target revealed an RMSD (root mean square deviation) 0.606 Å, which confirms that the ligand binds to the same pocket and assures the dependability of parameters of docking.

## 4. Conclusions

The results presented in this paper demonstrate the beneficial impact of LC-HRESIMS profiling when combined with bioassay-guided drug research from marine invertebrates to accelerate the commonly long processes of identifying an active metabolites by successive isolation from crude extracts. Dereplication experiments focused on chemotaxonomic sorting helped identify putatively active metabolites, while structural elucidation of the isolated compounds, using both HRMS and NMR, verified the hits. The bioactive extract metabolomic profiling indicated the existence of various secondary metabolites, primarily fatty acids, phenolic diterpenes, and triterpenes. Thus, bioassay-guided isolation coupled to LC-HRESIMS metabolomic profiling of the Red Sea cucumber *Holothuria spinifera* led to the characterization of four compounds: three new cerebrosides (compounds **1**, **2**, and **3**), and cholesterol sulfate (compound **4**), marking the first report of it in the sea cucumber species *Holothuria spinifera*. Compounds **2** and **3** showed larger cytotoxic effects against MCF-7 cancer cells, with IC_50_ values of 8.13 and 8.27 µM, respectively. Compound **1** displayed a slightly lower activity, with an IC_50_ value of 13.83 µM, while the steroid **4** was the least active, displaying an IC_50_ value of 35.56 µM. The cytotoxicities of compounds **1**, **2**, **3**, and **4** were compared to that of the standard drug doxorubicin with an IC_50_ value of 8.64 µM, which was used as a positive control. Finally, the obtained findings were confirmed by docking studies, which demonstrated the abilities of the four compounds to fit to the binding site of the SET oncoprotein and the inhibitor of PP2A. We assume that in vivo studies on these remarkable metabolites will be of impact to the potential development of new antineoplastic drugs.

## Figures and Tables

**Table 1:** ^1^H (400 MHz) and ^13^C NMR (100 MHz) data for the new compounds **1**, **2**, and **3** in C_5_D_5_N.

1			2			3		
Position	*δ_H_* (mult., *J*_Hz_)	*δ_C_*	Position	*δ_H_* (mult., *J*_Hz_)	*δ_C_*	Position	*δ_H_* (mult., *J*_Hz_)	*δ_C_*
**1**	4.26, m 4.76, dd (4, 12)	70.5	**1**	4.21, m 4.75, m	70.0	**1**	4.34, m 4.61, m	70.2
**2**	4.82, m	54.9	**2**	4.79, m	54.6	**2**	5.30, br,m	51.4
**3**	4.82, m	72.6	**3**	4.79, m	72.5	**3**	4.35, m	74.9
**4**	5.48, m	132.2	**4**	5.96, m	131.9	**4**	4.20, m	72.1
**5**	5.96, m	132.6	**5**	5.96, m	131.1	**5**	1.70, m	33.8
**6**	2.04, m	32.6	**6**	2.02, m	32.8	**6**	1.26	29.4
**7**	1.25	32.0	**7**	1.69, m	31.9	**7**	1.26	30.1
**8–15**	1.25	29.6	**8**	1.25	31.9	**8**	1.26	30.1
**16**	0.85, t (6.8)	14.1	**9–19**	1.25	29.7	**9–19**	1.26	29.7
**1’**	-	173.4	**20**	0.85, t (8.0)	14.1	**20**	0.87, t (6.8)	14.0
**2’**	4.79, m	72.6	**1’**	-	175.7	**1’**	-	175.4
**3’**	2.04, m	32.0	**2’**	4.56, m	72.4	**2’**	4.74, t (8.0)	72.2
**4’**	1.25	28.0	**3’**	2.18, m	32.94	**3’**	1.70, m	33.8
**5’**	1.25	27.6	**4’–13’**	1.25	29.7	**4’–12’**	1.26	29.7
**6’–16’**	1.25	29.6	**14’**	2.02, m	31.9	**13’**	2.12, m	31.9
**17’**	0.85, t (6.8)	14.1	**15’**	5.25, m	129.9	**14’**	5.30, m	129.9
**NH**	8.37, d (8.0)	-	**16’**	5.49, m	132.3	**15’**	5.50, m	130.0
**1’’**	4.53, d (8.0)	105.9	**17’**	2.02, m	31.9	**16’**	2.12, m	31.9
**2’’**	4.06, t (8.0)	75.2	**18’–22’**	1.25	29.7	**17’–21’**	1.26	29.7
**3’’**	4.22, m	78.5	**23’**	0.85, t (6.8)	14.1	**22’**	0.87, t (6.8)	14.0
**4’’**	4.22, m	71.5	**NH**	8.37, d (8.0)	-	**NH**	8.60, d (8.0)	-
**5’’**	3.94, m	78.5	**1’’**	4.98, d (8.0)	105.6	**1’’**	4.97, d (8.0)	105.1
**6’’**	4.37, m 4.75, dd (4.0, 12.0)	62.6	**2’’**	4.02, t (8.0)	75.0	**2’’**	4.02, m	75.5
			**3’’**	4.20, m	78.4	**3’’**	4.55, m	78.1
			**4’’**	4.20, m	71.4	**4’’**	4.74, m	71.1
			**5’’**	3.90, m	78.5	**5’’**	3.88, br,m	78.3
			**6’’**	4.36, dd (12.0, 4.0) 4.56, m	62.5	**6’’**	4.35, dd (4.0, 8.0) 4.53, m	62.3

“Overlapped signals are listed without multiplicity.”

**Table 2:** Dereplicated metabolites reported to occur in *Holothuria spinifera*.

	RT (min)	MZmine ID	Molecular Weight	Name	Source	Reference
1	12.29	171	314.2249	Aureol	Porifera *Hyrtios* sp.	[33]
2	10.87	122	314.2252	Epichromazonarol	*Dictyopteris undulata*	[34]
3	5.63	96	330.2398	Plakortether E	Porifera *Plakortis simplex*	[29]
4	11.23	109	332.2338	Diemenensin A	*Siphonaria diemenensis*	[35]
5	10.91	154	332.2353	Yahazunol	Algae *Dictyopteris undulata*	[36]
6	0.68	168	342.1149	Isomaltose	*Bacillus polymxa* and *Streptomyces* spp.	[37]
7	4.85	131	352.0208	Thelephoric acid	Basidiomycete *Polyozellus multiflex*	[31]
8	12.63	144	372.1592	Fellutanine	*Penicillium fellutanum*	[38]
9	11.78	75	374.2976	(22*E*)-Ergosta-2,5,7,9(11),14,22-hexaene	*Suillus luteus*	[39]
10	11.58	115	426.3135	Stoloniferone O	*Clavularia viridis*	[30]
11	9.25	24	453.2860	Terpendole F	*Albophoma yamanashiensis*	[32]
12	10.29	20	506.3224	Iriomoteolide-1b	Marine *Amphidinium* species	[40]
13	6.72	155	508.2678	Briareolate ester D	Cnidaria *Briareum asbestinum*	[41]

**Table 3:** IC_50_ values of compounds **1**, **2**, **3**, and **4** on the MCF-7 breast cancer cell line.

Compound No.	IC_50_ (µM)
**1**	13.83 ± 0.06 * µM
**2**	8.13 ± 0.01 µM
**3**	8.27 ± 0.03 µM
**4**	35.56 ± 0.12 µM
Doxorubicin	8.64 ± 0.02 µM

Each data point represents the mean ± SD of three independent experiments (significant differences at *p* < 0.05). * The expressed µM value is for spiniferoside A***2*** (**1b**) as the major component in **1**.

**Table 4:** Molecular docking studies.

Compound No.	Binding Energy Score	Average Number of Poses per Run
**1a**	−12.493	30
**1b**	−10.518	30
**1c**	−12.586	30
**2**	−11.482	30
**3**	−9.854	30
**4**	−7.238	30

Each score shown is the mean of three consecutive runs. The docking method was validated by a successful pose-retrieval docking experiment of the ligand (score: −8.689).

## Supplementary Materials

The following are available online at https://www.mdpi.com/1660-3397/18/8/405/s1, Figures S1−S15: LC-HRESIMS, ^1^H NMR, ^13^C NMR and HPLC purification of compound 1, Figures S16–S24: LC-HRESIMS, ^1^H NMR, ^13^C NMR of compound 2, Figure S25: LC-HRESIMS for *α*-hydroxy fatty acid methyl ester after hydrolysis of compound 2, Figure S26: GC-MS analysis of fatty acid methyl ester carried out after oxidation of *α*-hydroxy fatty acid methyl ester compound 2, Figures S27–31: LC-HRESIMS, ^1^H NMR and ^13^C NMR of compound 3, Figure S32: LC-HRESIMS for *α*-hydroxy fatty acid methyl ester after hydrolysis of compound 3, Figure S33: GC-MS analysis of fatty acid methyl ester carried out after oxidation of *α*-hydroxy fatty acid methyl ester of compound 3, Figures S34–S35: ^1^H NMR and ^13^C NMR of compound 4 and Figures S36–S40: Cytotoxicity of compounds 1, 2, 3, 4 and doxorubicin on MCF-7.
