# Supplementary material for: New Cytotoxic Cerebrosides from the Red Sea Cucumber Holothuria spinifera Supported by In-Silico Studies
Source: Mar Drugs. 2020 Aug 1;18(8):405. doi: 10.3390/md18080405 (PMC7460232; doi:10.3390/md18080405)
Supplement: Supplementary file 1 [file marinedrugs-18-00405-s001.pdf]

New Cytotoxic Cerebrosides from the Red Sea Cucumber *Holothuria spinifera* Supported by *In-Silico* Studies

Reda F. A. Abdelhameed [1,+], Enas E. Eltamany [1+], Dina M. Hal [1], Amany K. Ibrahim [1], Asmaa M. AboulMagd [2], Tarfah Al-Warhi [3], Khayrya A. Youssif [4], Adel M. Abd El-kader [5,6], Hashim A Hassanean [1], Shaimaa Fayez [7,8], Gerhard Bringmann [7,*], Safwat A. Ahmed [1,*], Usama Ramadan Abdelmohsen [5,9]

[1] Department of Pharmacognosy, Faculty of Pharmacy, Suez Canal University, Ismailia, Egypt
[2] Department of Pharmaceutical Chemistry, Faculty of Pharmacy, Nahda University, Beni Suef, Egypt
[3] Department of Chemistry, College of Science, Princess Nourah bint Abdulrahman University, Riyadh, Saudi Arabia
[4] Department of Pharmacognosy, Faculty of Pharmacy, Modern University for Technology and Information, Cairo, Egypt
[5] Department of Pharmacognosy, Faculty of Pharmacy, Deraya University, New Minia 61111, Egypt
[6] Department of Pharmacognosy, Faculty of Pharmacy, Al-Azhar University, Assiut 71524, Egypt
[7] Institute of Organic Chemistry, University of Würzburg, Am Hubland, 97074 Würzburg, Germany
[8] Department of Pharmacognosy, Faculty of Pharmacy, Ain-Shams University, 11566 Cairo, Egypt
[9] Department of Pharmacognosy, Faculty of Pharmacy, Minia University, 61519, Minia, Egypt
[+] Equal contribution
***** Correspondence: S.A.A., Phone: (+20) 010-92638387, Fax: (+20) 064-3230741, email: safwat_aa@yahoo.com; G.B., Phone: (+49) 0931-3185323, Fax: (+49) 0931-3184755, email bringmann@chemie.uni-wuerzburg.de

Received: 30 June 2020; Accepted: 23 July 2020; Published: July 2020

Table of Contents

**Figure S1:**	**LC-HRESIMS of Compound 1 (+MS)…………………………………………………………………………**	**4**
**Figure S2:**	^{1}H NMR spectrum of compound **1** in (C_5D_5N, 400 MHz)…………………………………………………	**5**
**Figure S3:**	Partial expansion of the ^{1}H NMR spectrum of compound **1** in (C_5D_5N, 400 MHz)…………………………..	**6**
**Figure S4:**	Partial expansion of the ^{1}H NMR spectrum of compound **1** in (C_5D_5N, 400 MHz)…………………………...	**7**
**Figure S5:**	^{13}C NMR spectrum of compound **1** in (C_5D_5N, 100 MHz)...	**8**
**Figure S6:**	Partial expansion of the ^{13}C NMR spectrum of compound **1** in (C_5D_5N, 100 MHz)...	**9**
**Figure S7:**	Partial expansion of the ^{13}C NMR spectrum of compound **1** in (C_5D_5N, 100 MHz)…………………………...	**10**
**Figure S8:**	Partial expansion of the ^{13}C NMR spectrum of compound **1** in (C_5D_5N, 100 MHz)………………………………...	**11**
**Figure S9:**	Chromatogram of semi-preparative HPLC purification of compound **1**……………………………......................	**12**
**Figure S10:**	Chromatogram of semi-preparative HPLC purification of spiniferoside A1 (**1a**) ………………………………….	**13**
**Figure S11:**	Chromatogram of semi-preparative HPLC purification of spiniferoside A2 (**1b**) ………………………………….	**14**
**Figure S12:**	Chromatogram of semi-preparative HPLC purification of spiniferoside A3 (**1c**)……………………………………..	**15**

**Figure S13:**	LC-HRESIMS for spiniferoside A1 (**1a**)……………………………………………………………………………..	**16**
**Figure S14:**	LC-HRESIMS for spiniferoside A2 (**1b**)………………………………………………………………………………	**17**
**Figure S15:**	LC-HRESIMS for spiniferoside A3 (**1c**)………………………………………………………………………………	**18**
**Figure S16:**	LC-HRESIMS of Compound **2** (+ MS)……………………………………………………………………………….	**19**
**Figure S17:**	^{1}H NMR spectrum of compound **2** in C_5D_5N (in 400 MHz)………………………………………………………..	**20**
**Figure S18:**	Partial expansion of the ^{1}H NMR spectrum of compound **2** (in C_5D_5N, 400 MHz)…………………………………	**21**
**Figure S19:**	Partial expansion of the ^{1}H NMR spectrum of compound **2** (in C_5D_5N, 400 MHz)………………………………….	**22**
**Figure S20:**	Partial expansion of the ^{1}H NMR spectrum of compound **2** (in C_5D_5N, 400 MHz)………………………………….	**23**
**Figure S21:**	^{13}C NMR spectrum of compound **2** (in C_5D_5N, 100 MHz)…………………………………………………………	**24**
**Figure S22:**	Partial expansion of the ^{13}C NMR spectrum of compound **2** (in C_5D_5N, 100 MHz)…………………………………..	**25**
**Figure S23:**	Partial expansion of the ^{13}C NMR spectrum of compound **2** (in C_5D_5N, 100 MHz)…………………………………	**26**
**Figure S24:**	Partial expansion of the ^{13}C NMR spectrum of compound **2** (in C_5D_5N, 100 MHz)………………………………….	**27**
**Figure S25:**	LC-HRESIMS for α-hydroxy fatty acid methyl ester after hydrolysis of compound **2**……………………………	**28**
**Figure S26:**	GC-MS analysis of fatty acid methyl ester carried out after oxidation of α-hydroxy fatty acid methyl ester (Compound 2)	**29**
**Figure S27:**	LC-HRESIMS of Compound **3** ………………………………………………………………………………	**30**
**Figure S28:**	^{1}H NMR spectrum of compound **3** (in C_5D_5N, 400 MHz)**…………………………………………………**	**31**
**Figure S29:**	Partial expansion of the ^{1}H NMR spectrum of compound **3** (in C_5D_5N, 400 MHz)…………………………………...	**32**
**Figure S30:**	^{13}C NMR spectrum of compound **3** (in C_5D_5N, 100 MHz)…………………………………………………………….	**33**
**Figure S31:**	Partial expansion of the ^{13}C NMR spectrum of compound **3** (in C_5D_5N, 100 MHz)…………………………………	**34**
**Figure S32:**	LC-HRESIMS for α-hydroxy fatty acid methyl ester after hydrolysis of compound **3**……………………………	**35**
**Figure S33:**	GC-MS analysis of fatty acid methyl ester carried out after oxidation of α-hydroxy fatty acid methyl ester………	**36**
**Figure S34:**	^{1}H NMR spectrum of compound **4** (in DMSO, 400 MHz)……………………………………………….............	**37**
**Figure S35:**	^{13}C NMR spectrum of compound **4** (in C_5D_5N, 100 MHz)…………………………………………………………….	**38**
**Figure S36:**	Cytotoxicity of compound **1** on MCF-7………………………………………………………………………	**39**
**Figure S37:**	Cytotoxicity of compound **2** on MCF-7……………………………………………………………………………..	**40**
**Figure S38:**	Cytotoxicity of compound **3** on MCF-7……………………………………………………………………………..	**41**
**Figure S39:**	Cytotoxicity of compound **4** on MCF-7……………………………………………………………………………..	**42**
**Figure S40:**	Cytotoxicity of Doxorubicin on MCF-7………………………………………………………………………	**43**

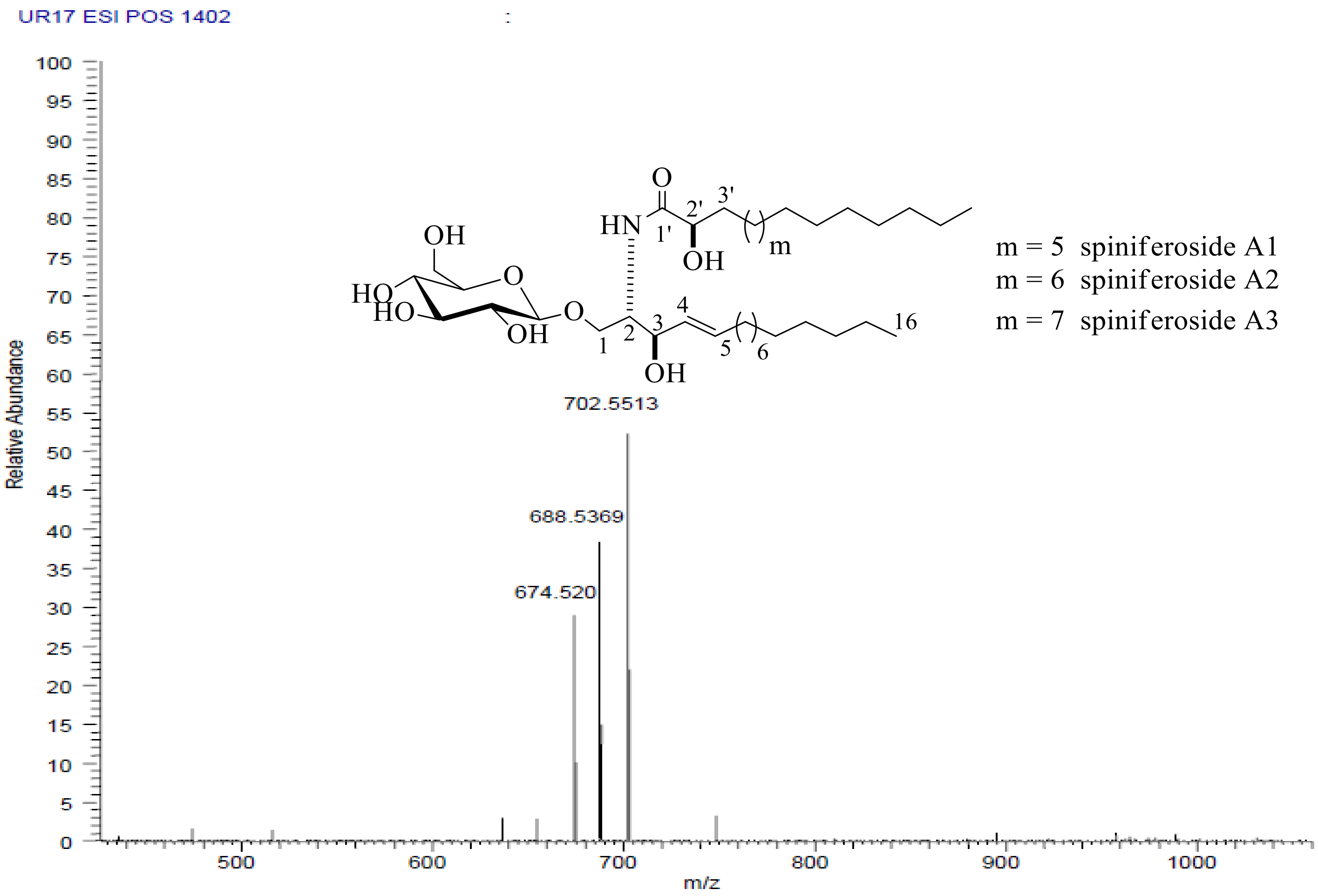

Figure S1. LC-HRESIMS of compound **1** (M+H)+.

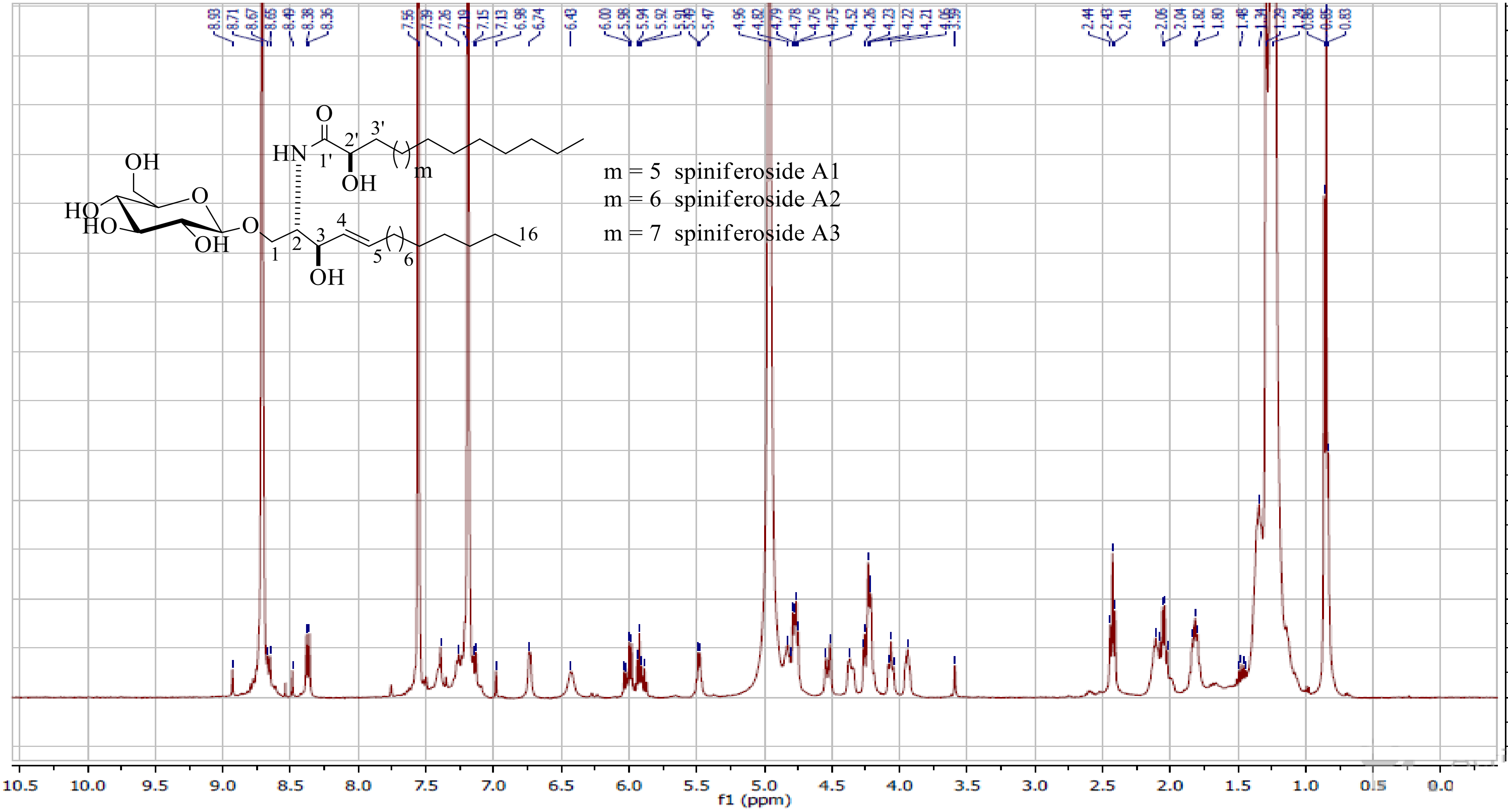

Figure S2. ^{1}H NMR spectrum of compound **1** (in C_5D_5N, 400 MHz).

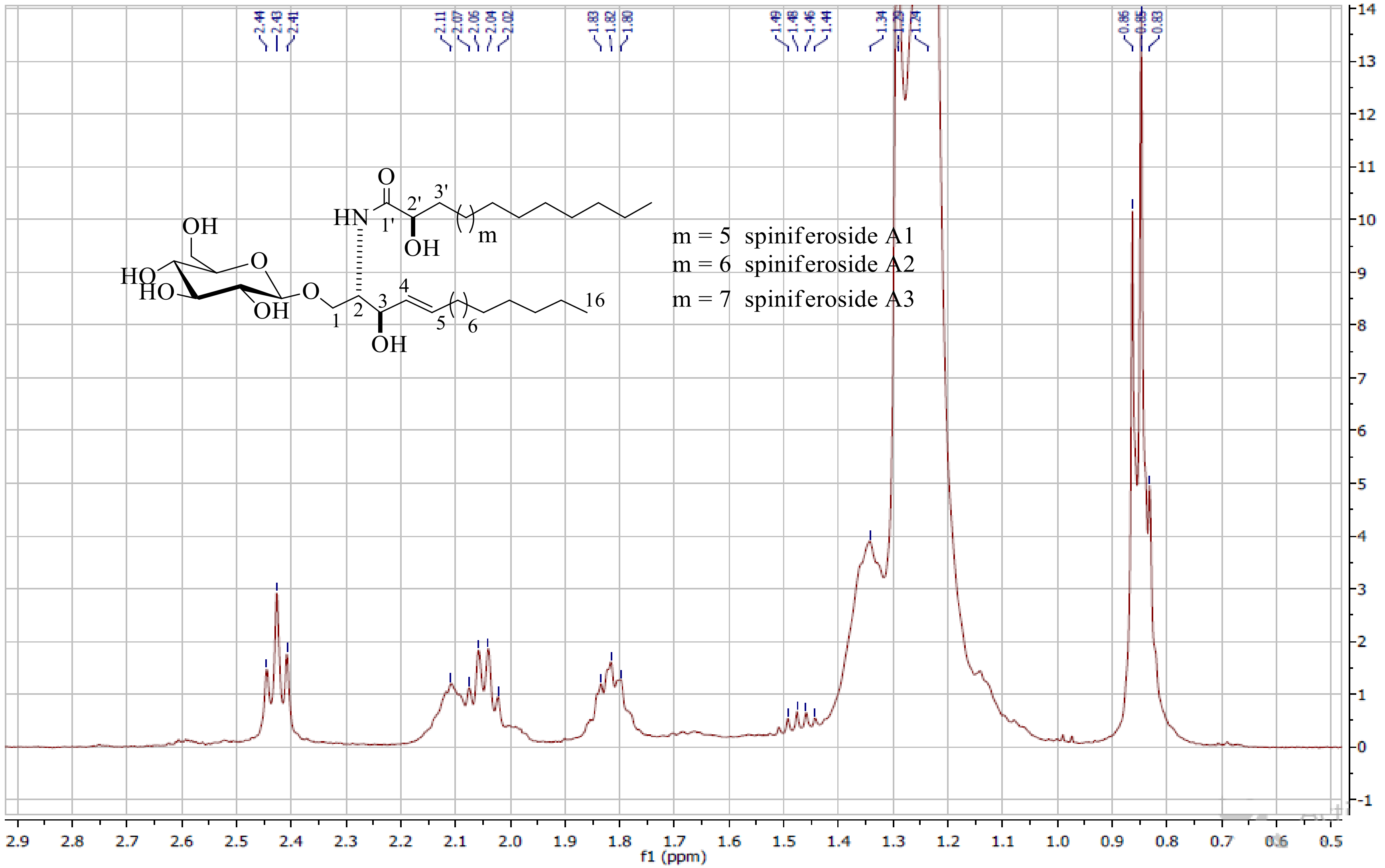

Figure S3. Partial expansion of the ^{1}H NMR spectrum of compound **1** (in C_5D_5N, 400 MHz).

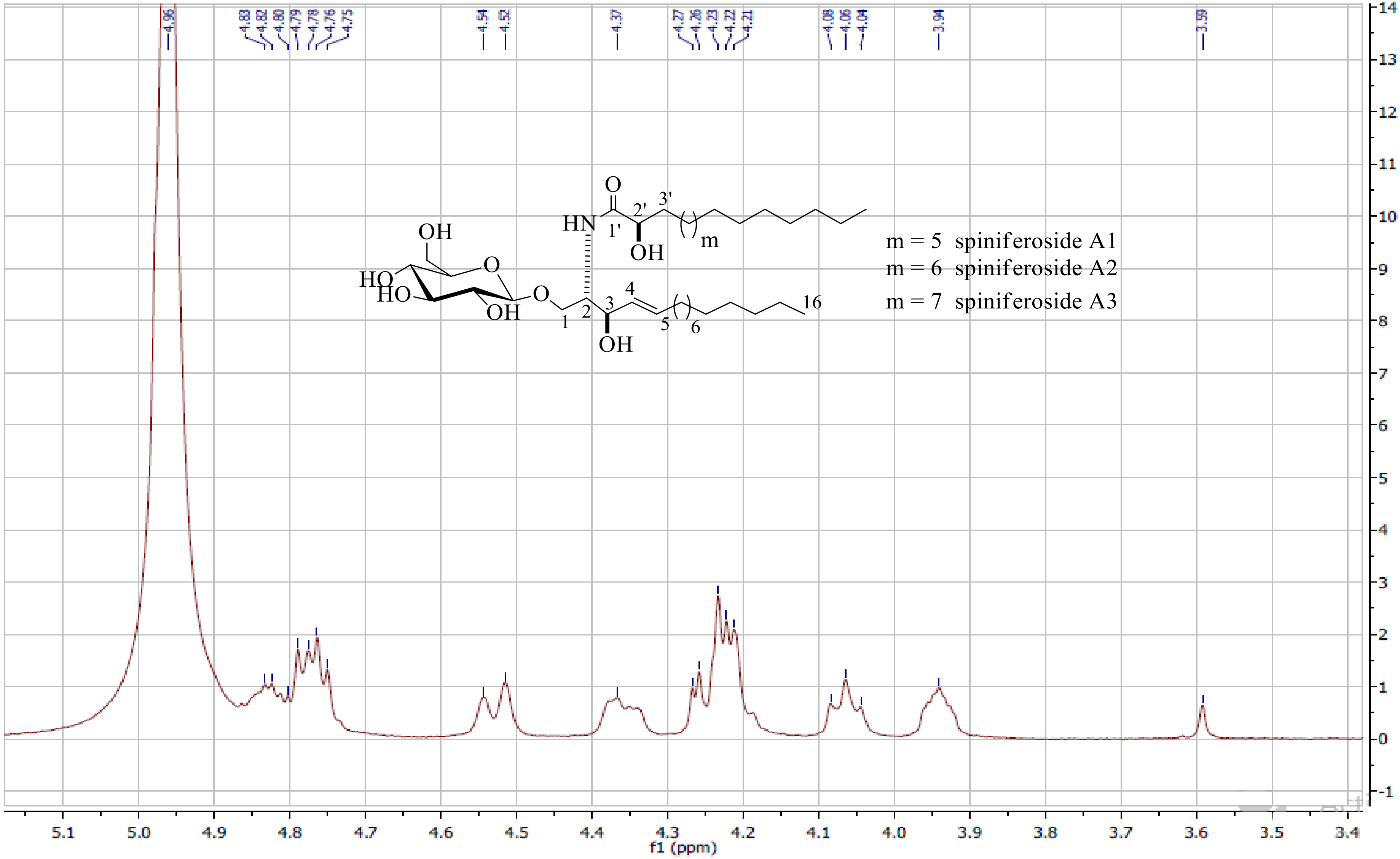

Figure S4. Partial expansion of the ¹H NMR spectrum of compound **1** (in C_5D_5N, 400 MHz).

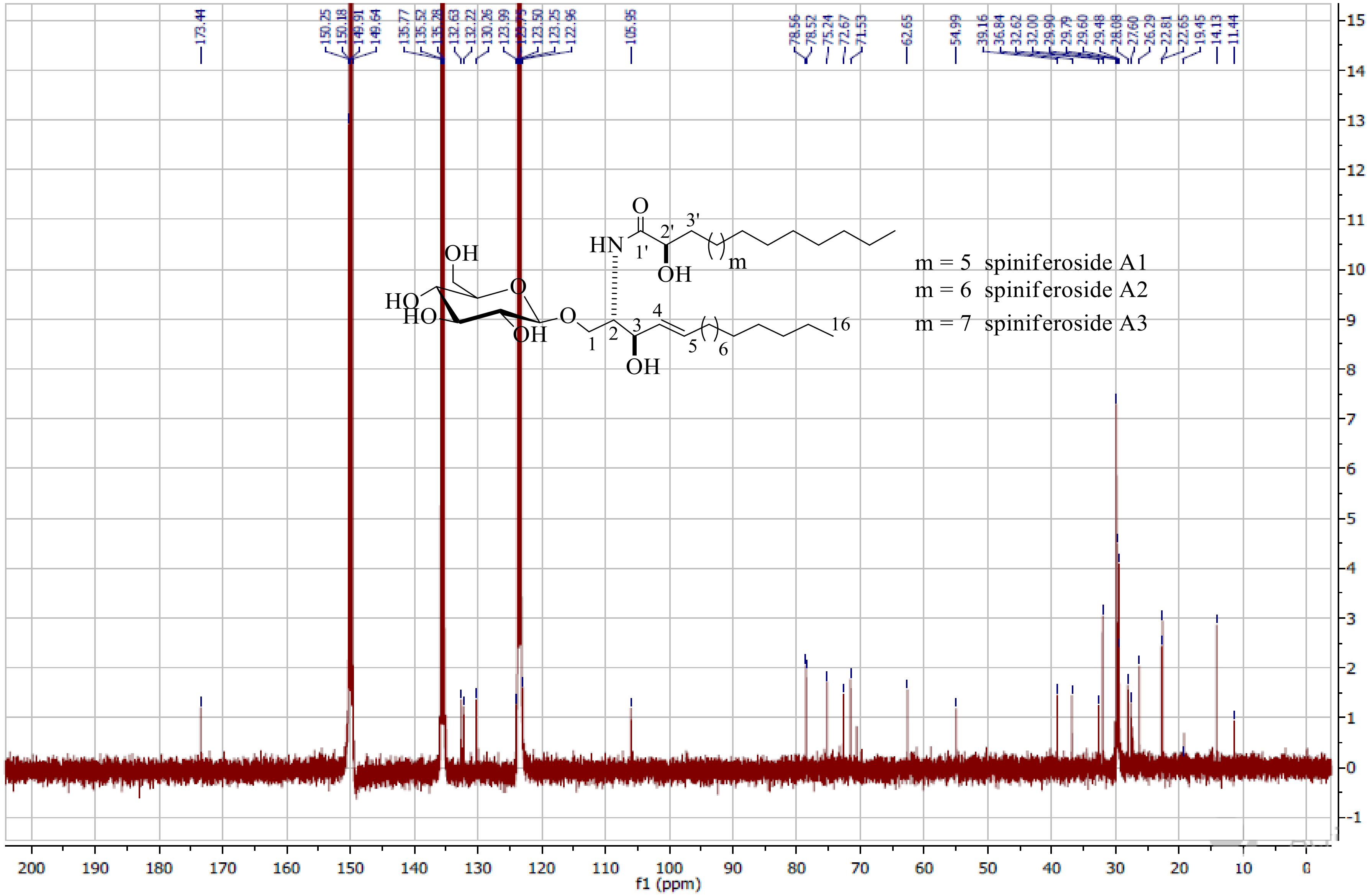

Figure S5. ^{13}C NMR spectrum of compound **1** (in C_5D_5N, 100 MHz).

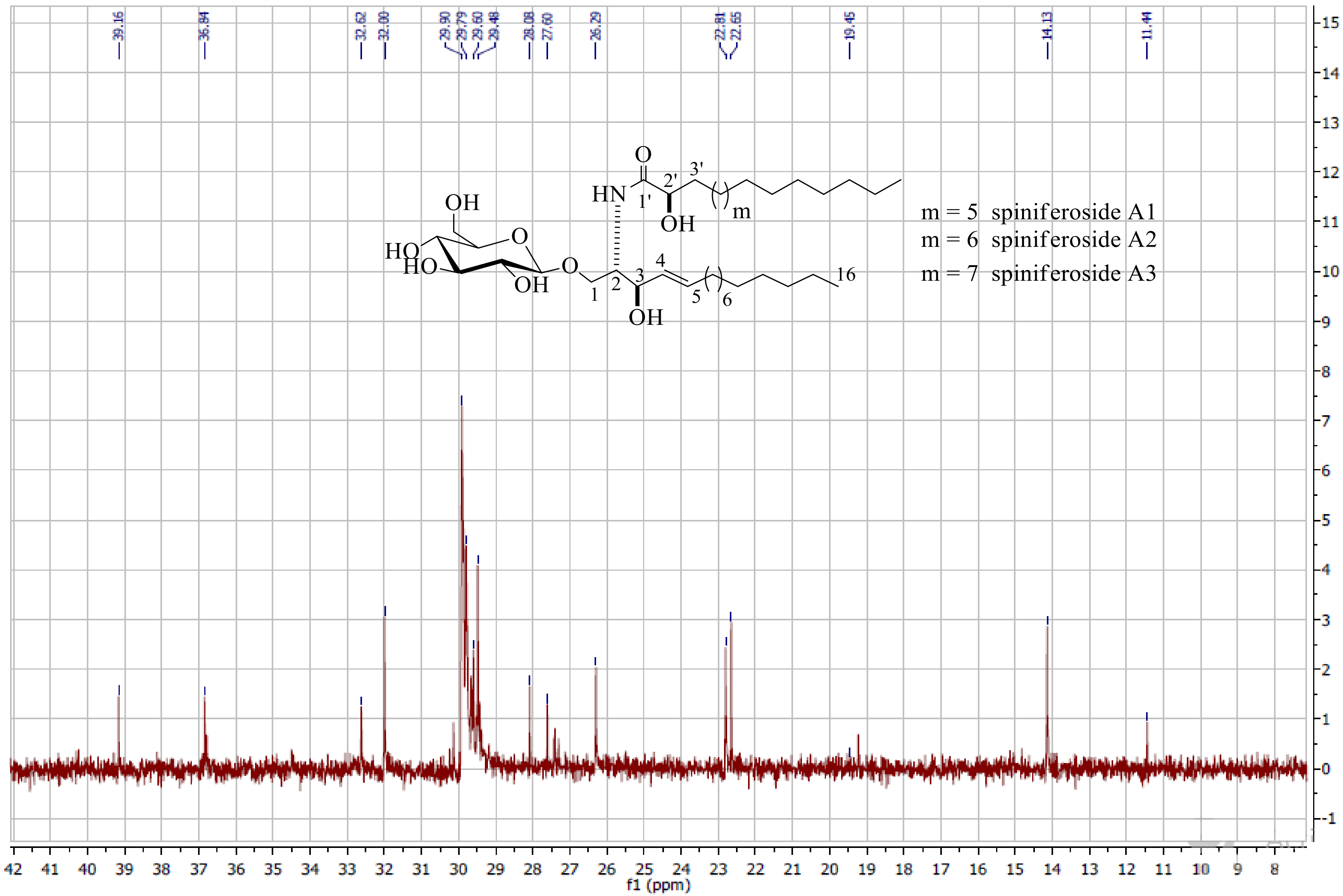

Figure S6. Partial expansion of the ^{13}C NMR spectrum of compound **1** (in C_5D_5N, 100 MHz).

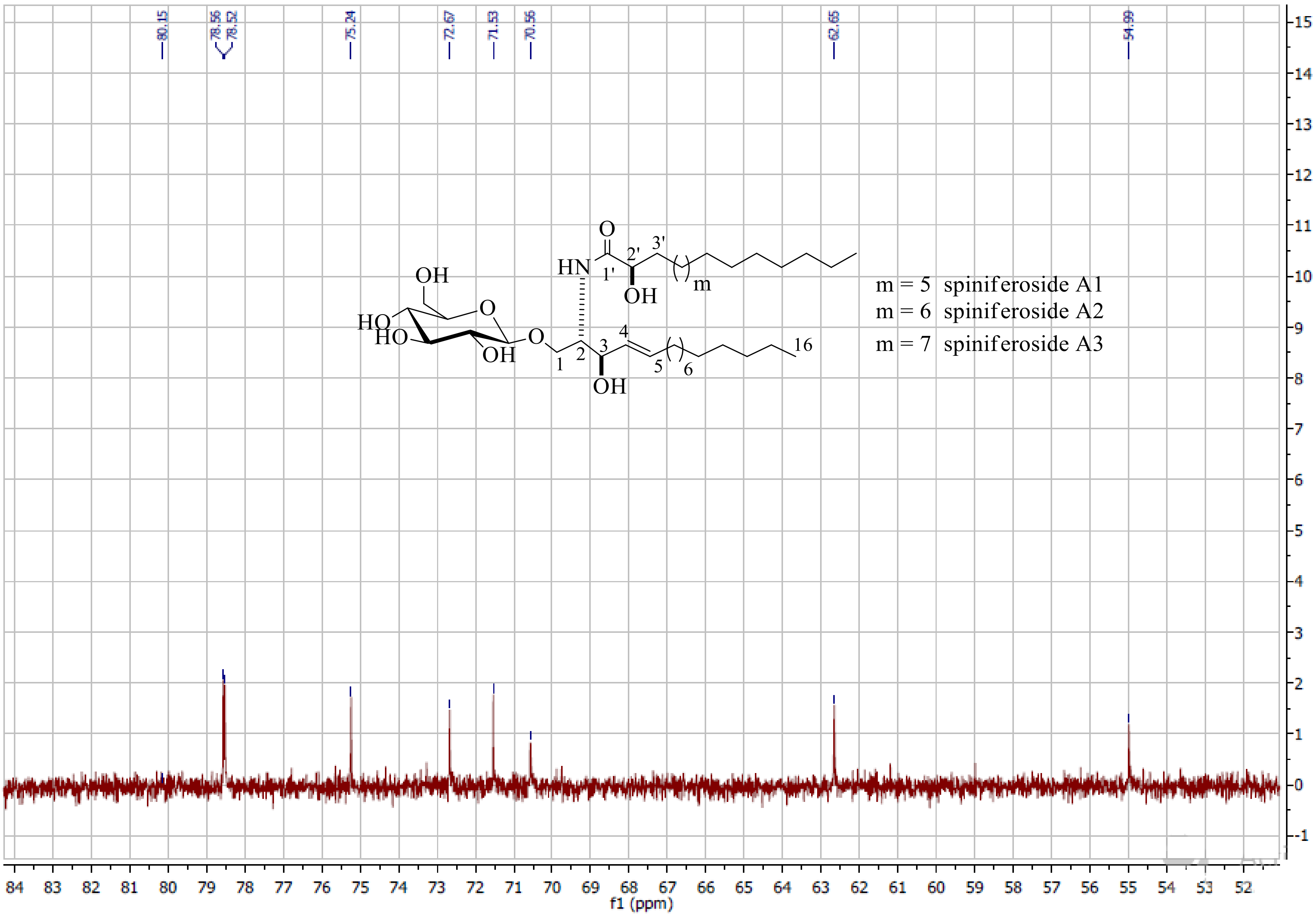

Figure S7. Partial expansion of the ^{13}C NMR spectrum of compound **1** (in C_5D_5N, 100 MHz).

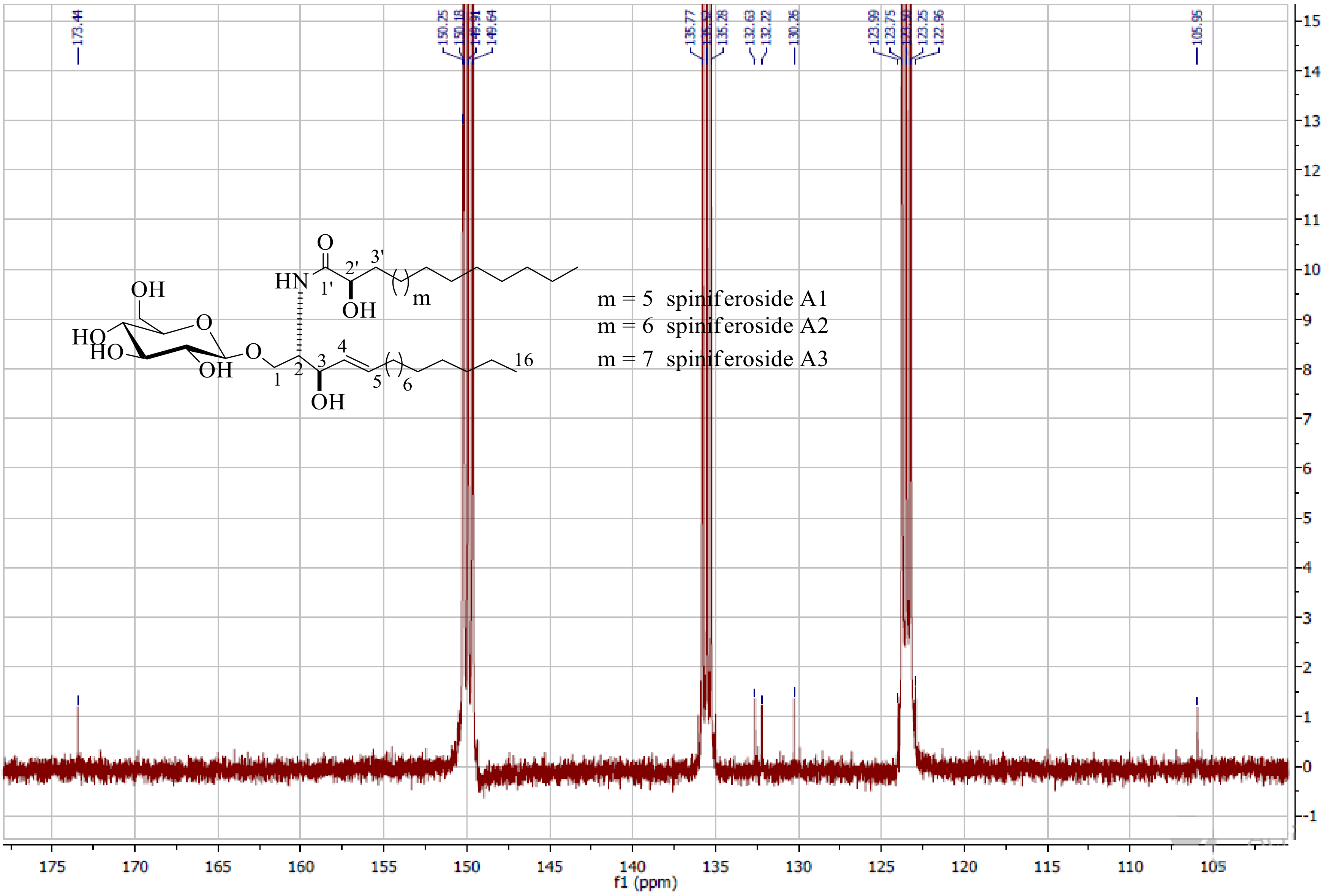

Figure S8. Partial expansion of the ^{13}C NMR spectrum of compound **1** (in C_5D_5N, 100 MHz).

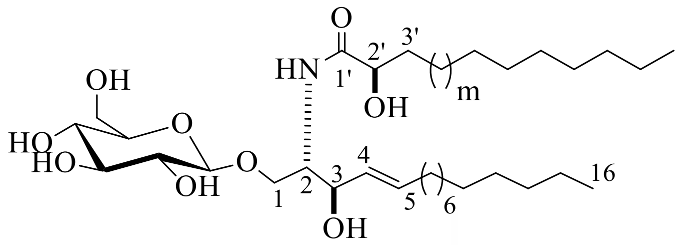

m = 5 spiniferoside A1
m = 6 spiniferoside A2
m = 7 spiniferoside A3

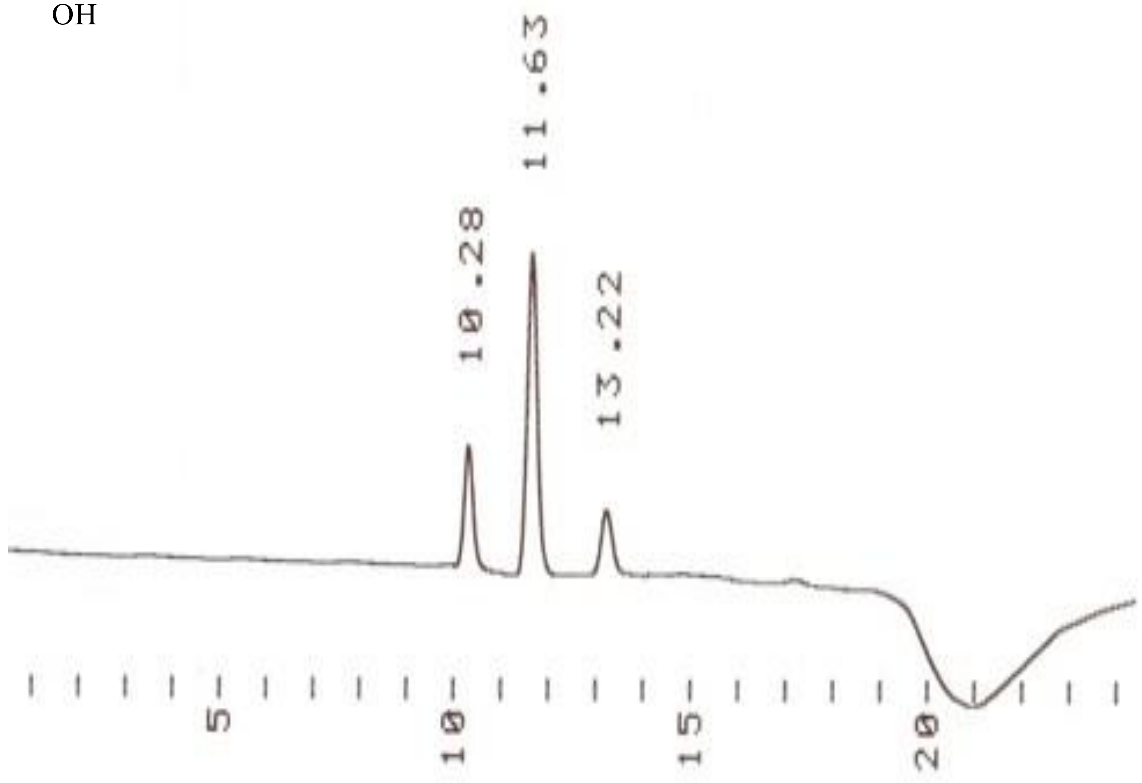

Figure S9. Chromatogram of semi-preparative HPLC purification of compound **1**.

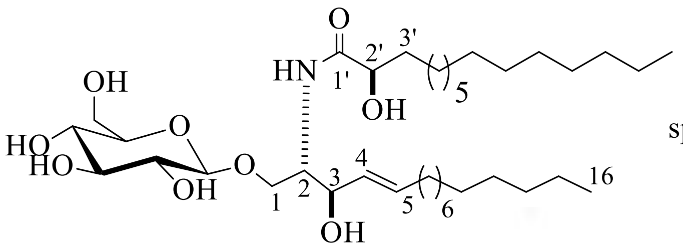

spiniferoside A1

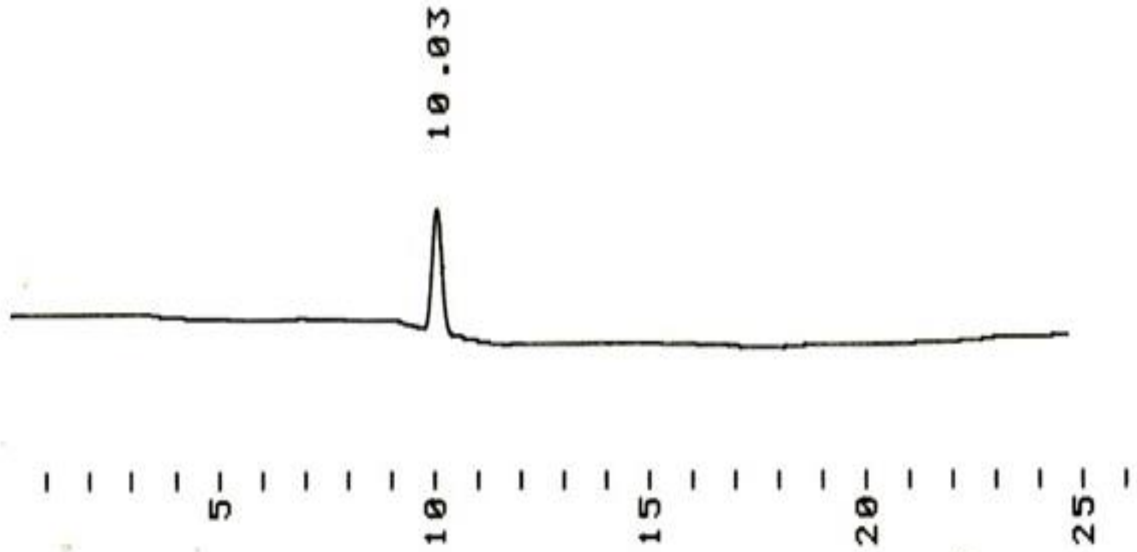

Figure S10. Chromatogram of semi-preparative HPLC purification of spiniferoside A1 (**1a**).

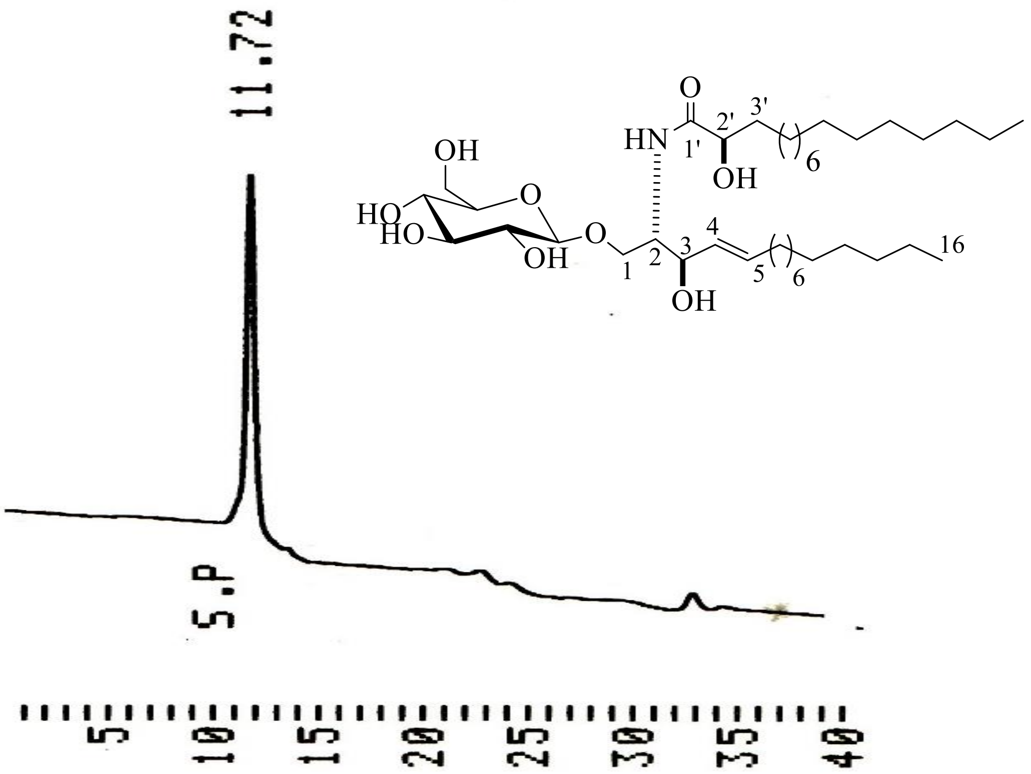

spiniferoside A2

Figure S11. Chromatogram of semi-preparative HPLC purification of spiniferoside A2 (**1b**).

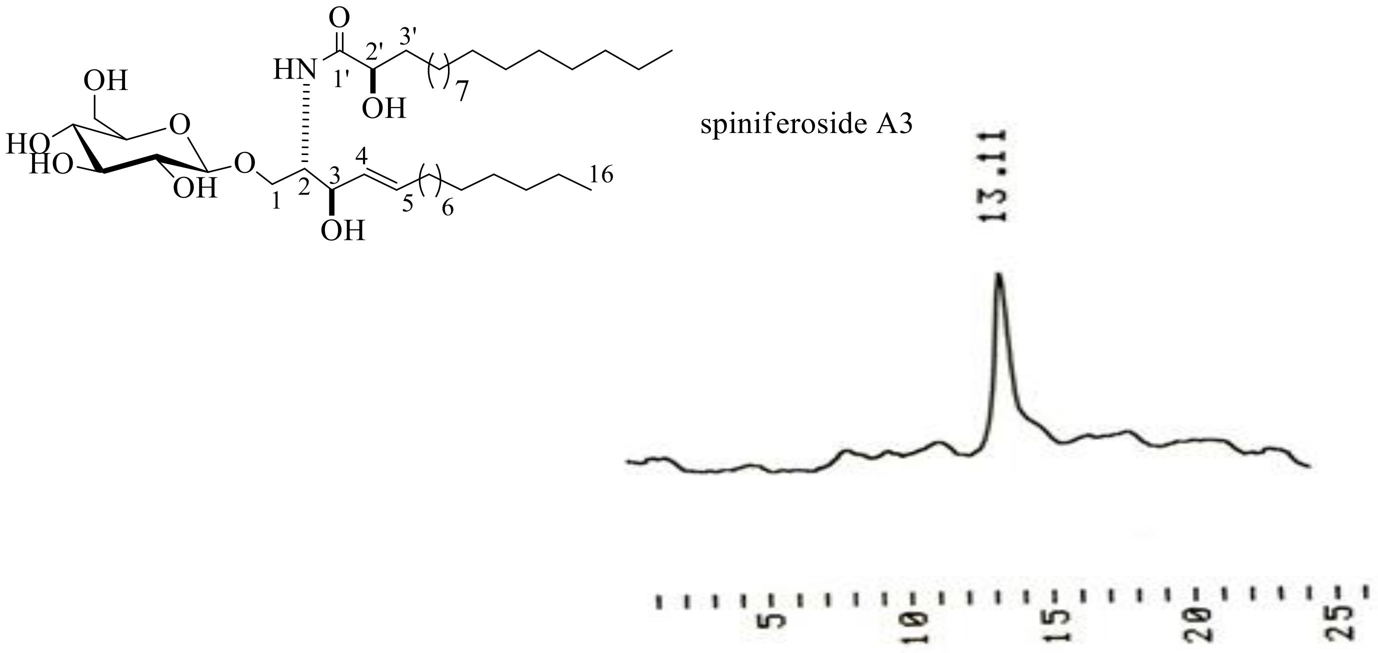

Figure S12. Chromatogram of semi-preparative HPLC purification of spiniferoside A3 (**1c**).

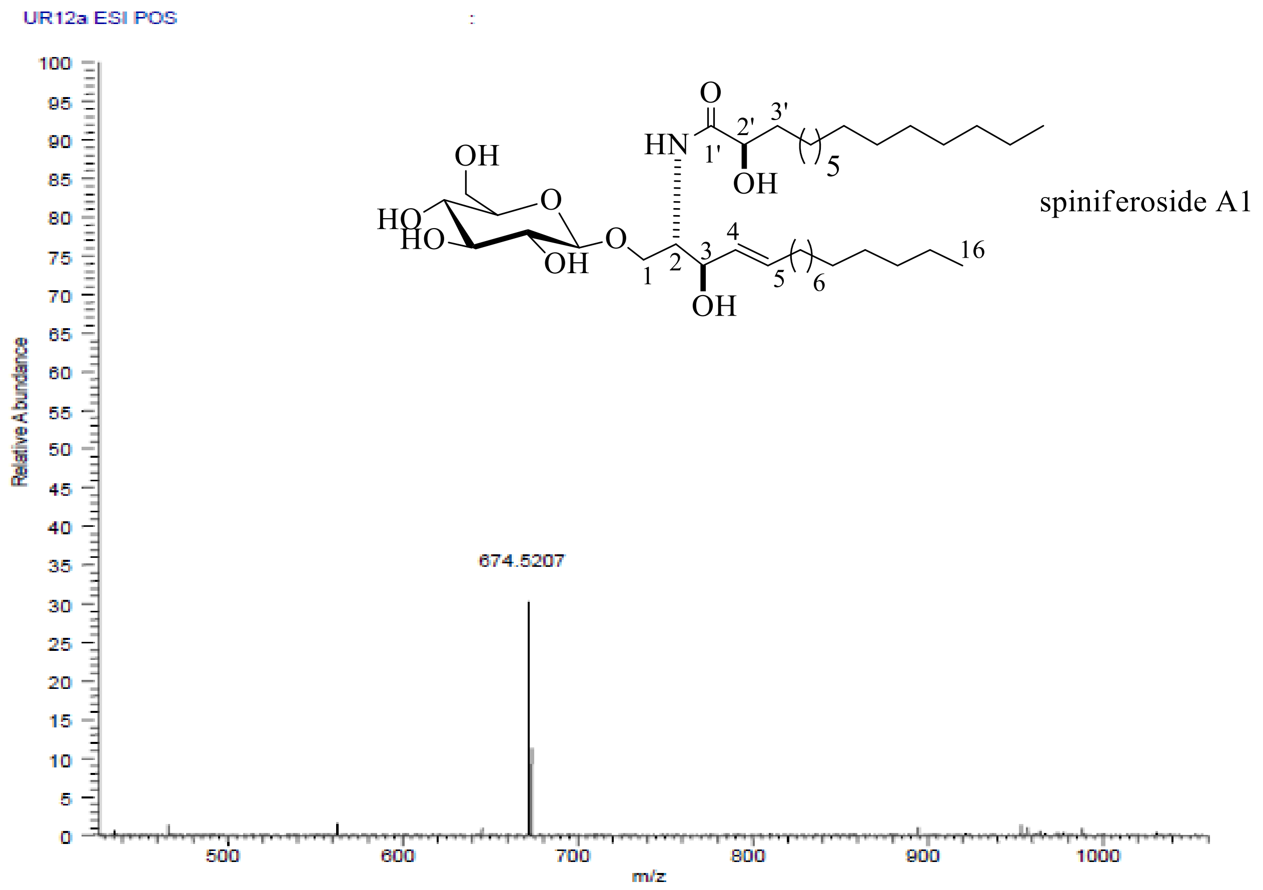

Figure S13. LC-HRESIMS for spiniferoside A1 (**1a**).

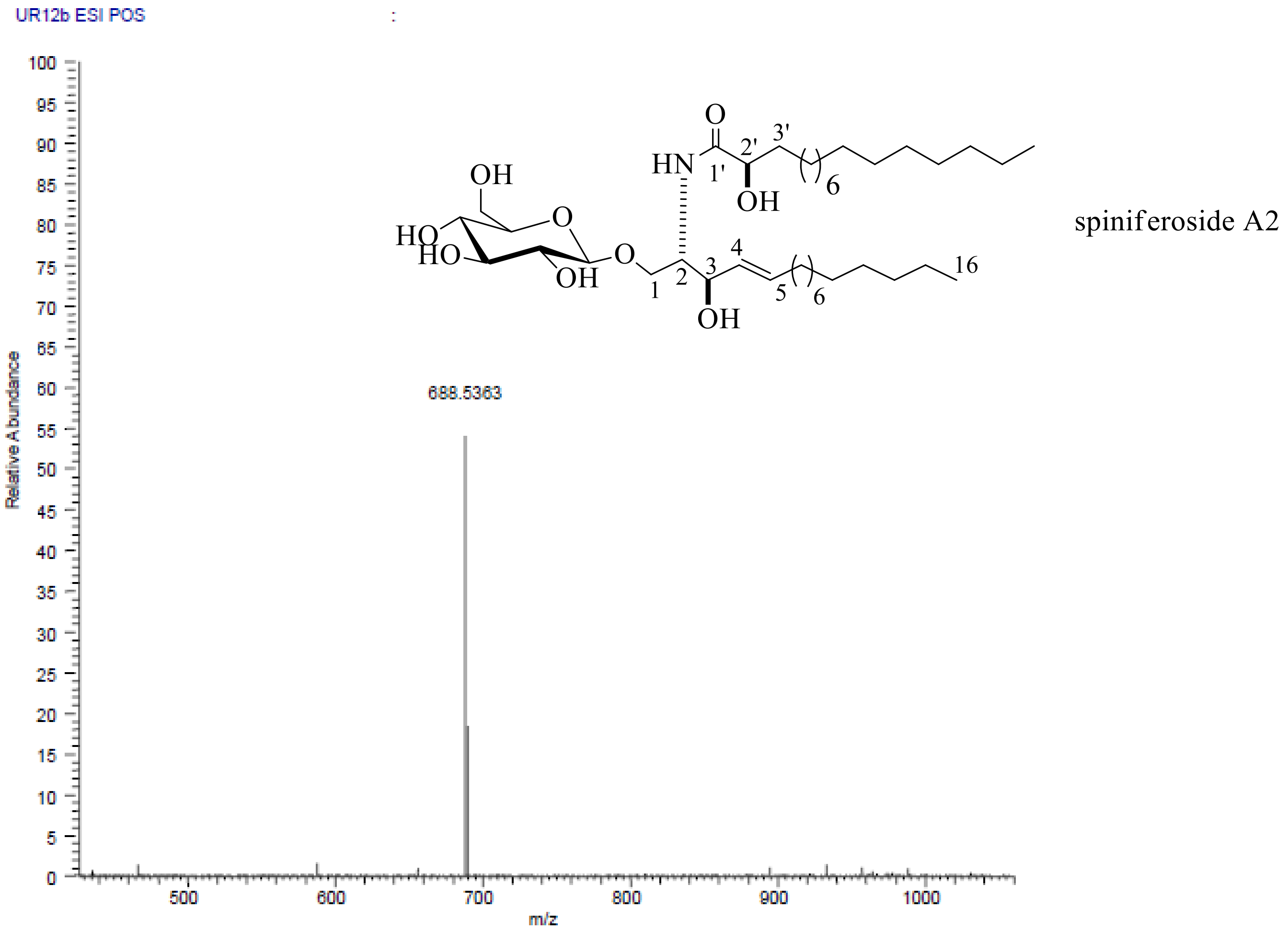

Figure S14. LC-HRESIMS for spiniferoside A2 (**1b**).

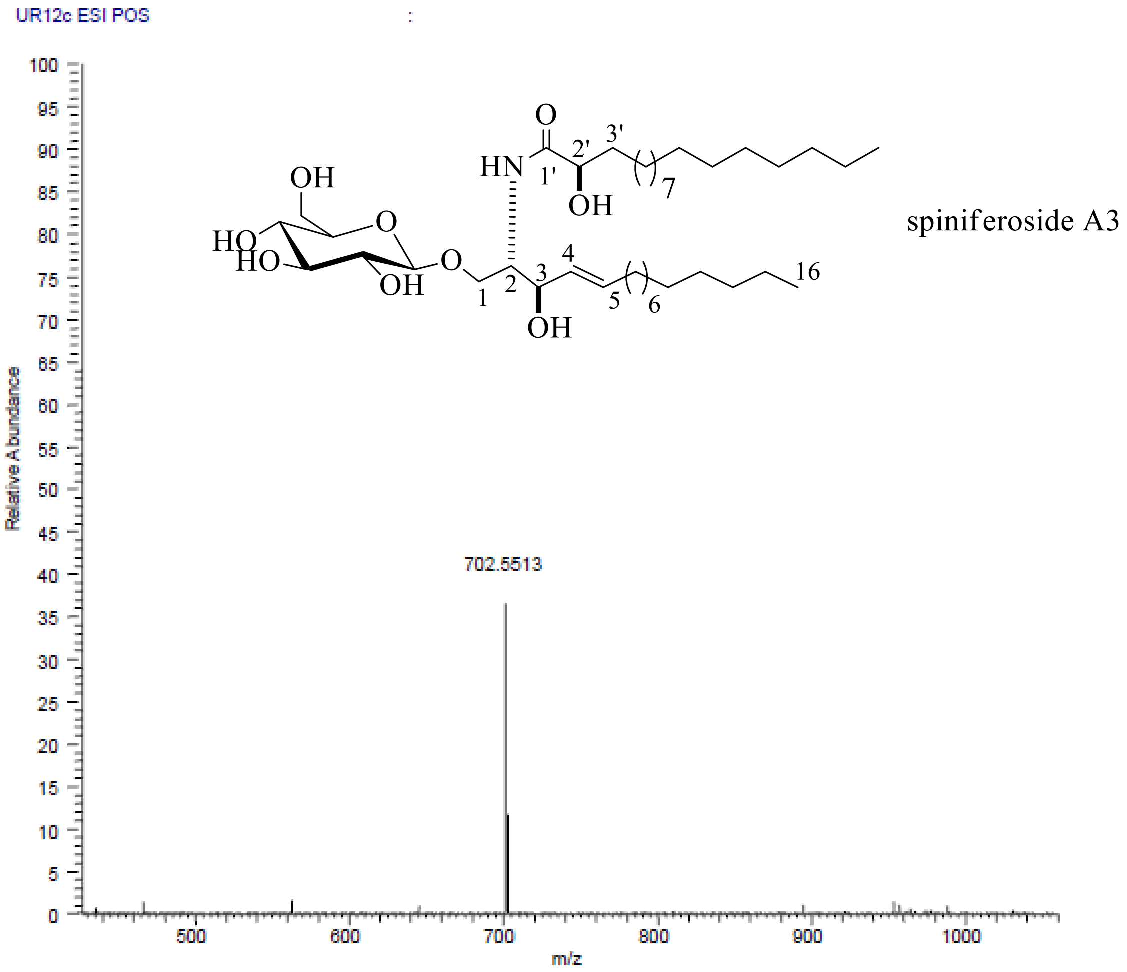

Figure S15: LC-HRESIMS for spiniferoside A3 (**1c**).

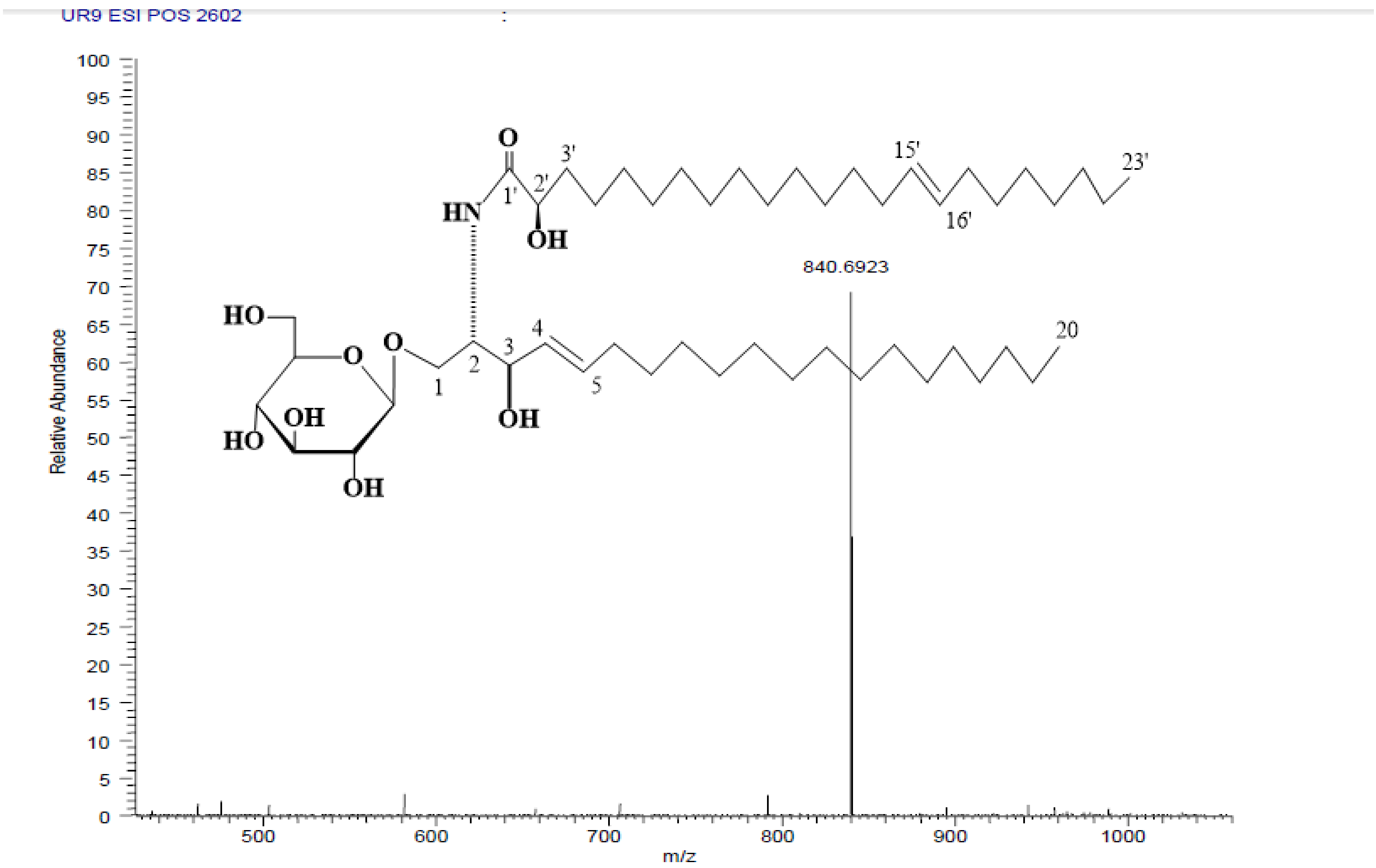

Figure S16. LC-HRESIMS of compound **2** $(M+H)^+$

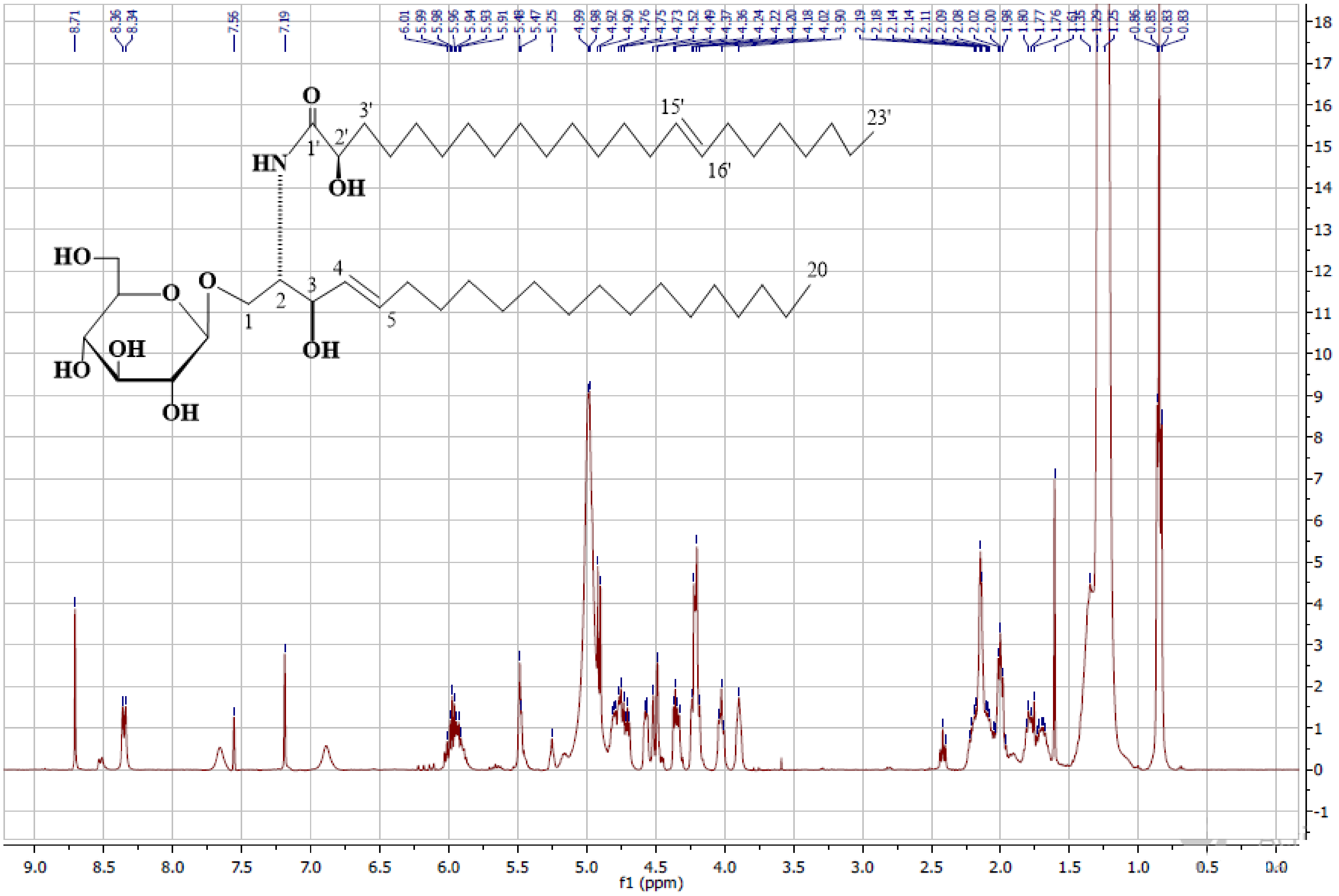

Figure S17. ^{1}H NMR spectrum of compound **2** (in C_5D_5N, 400 MHz).

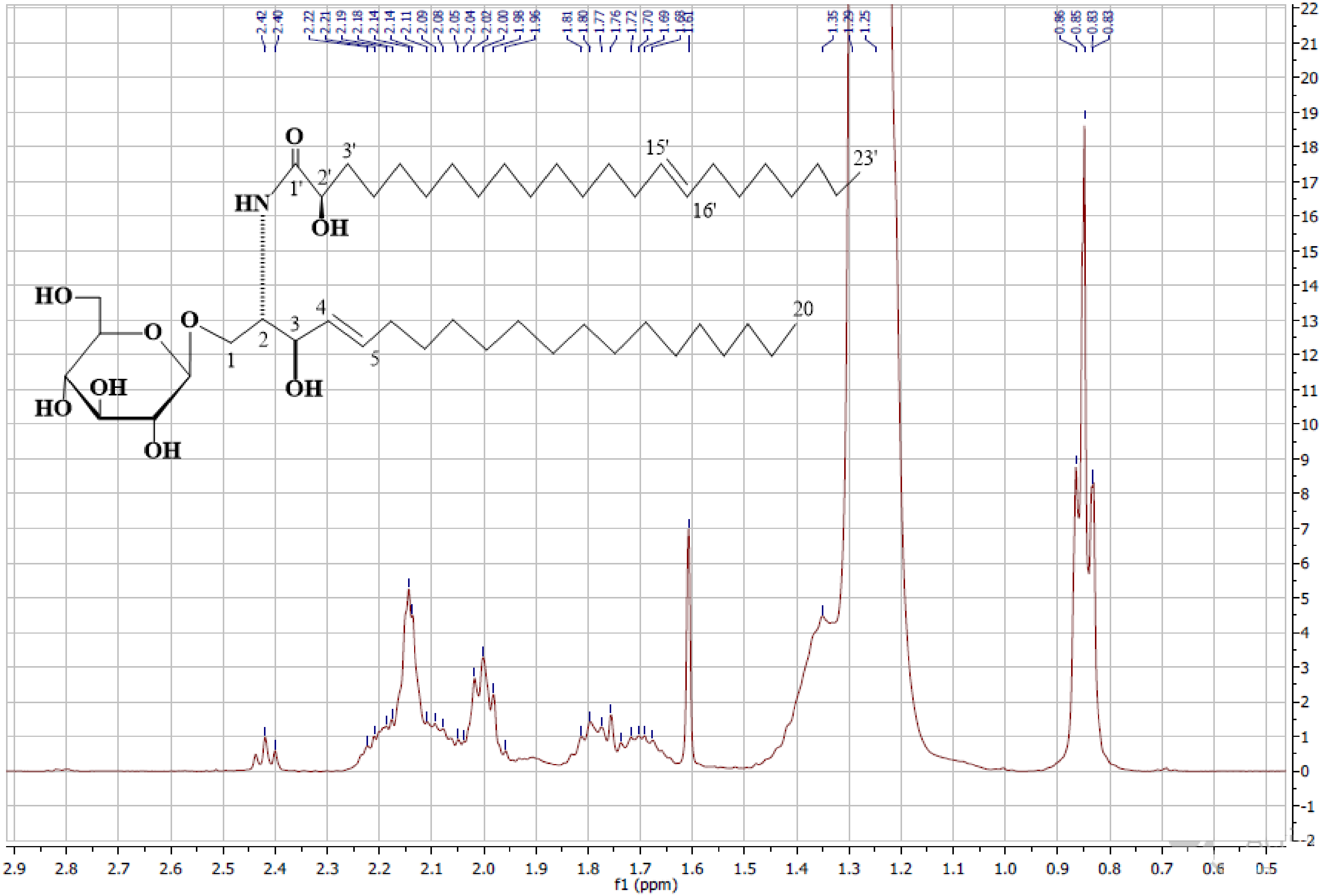

Figure S18. Partial expansions of the ^{1}H NMR spectrum of compound **2** (in C_5D_5N, 400 MHz).

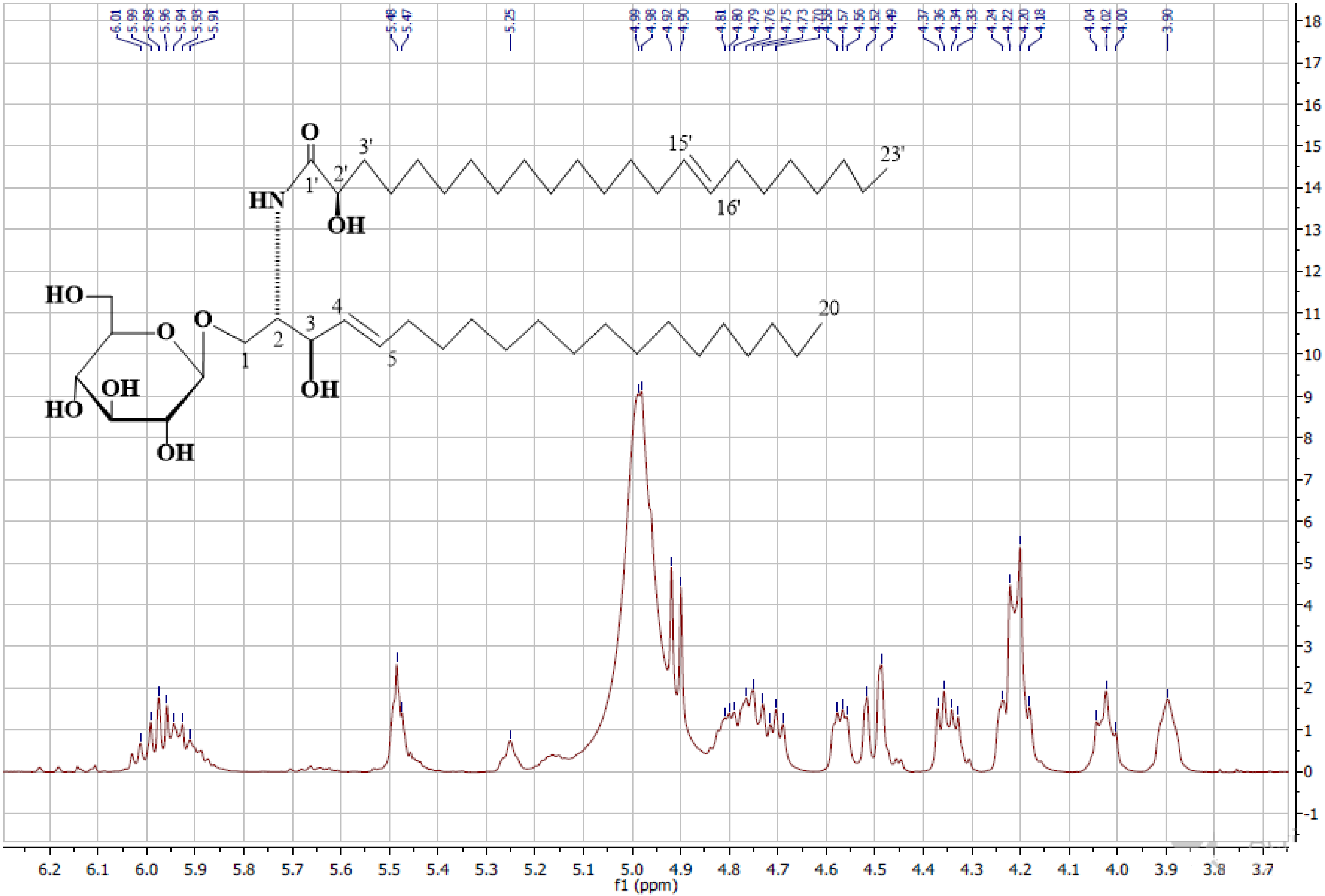

Figure S19. Partial expansions of the ^{1}H NMR spectrum of compound **2** (in C_5D_5N, 400 MHz).

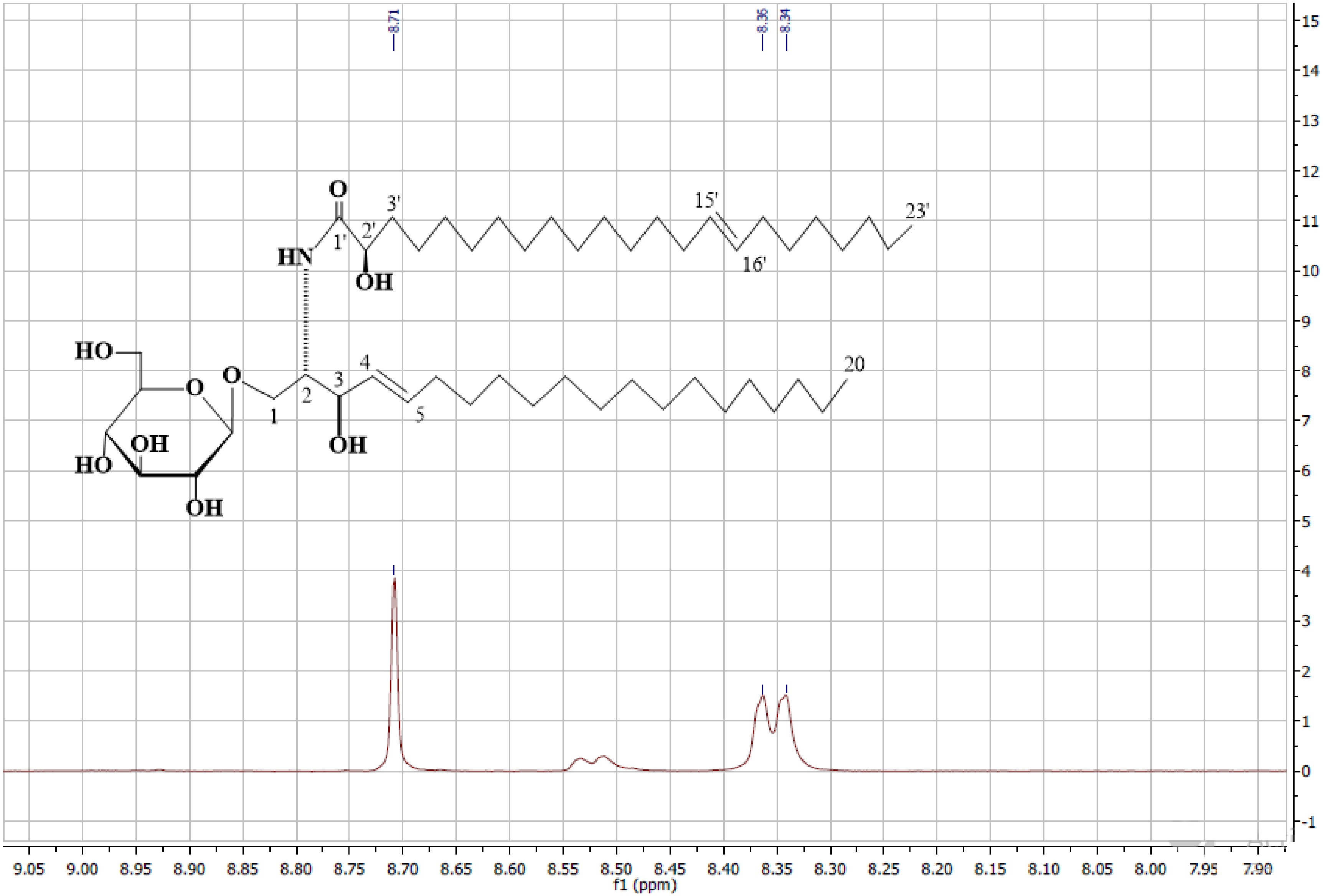

Figure S20. Partial expansions of the ^{1}H NMR spectrum of compound **2** (in C_5D_5N, 400 MHz).

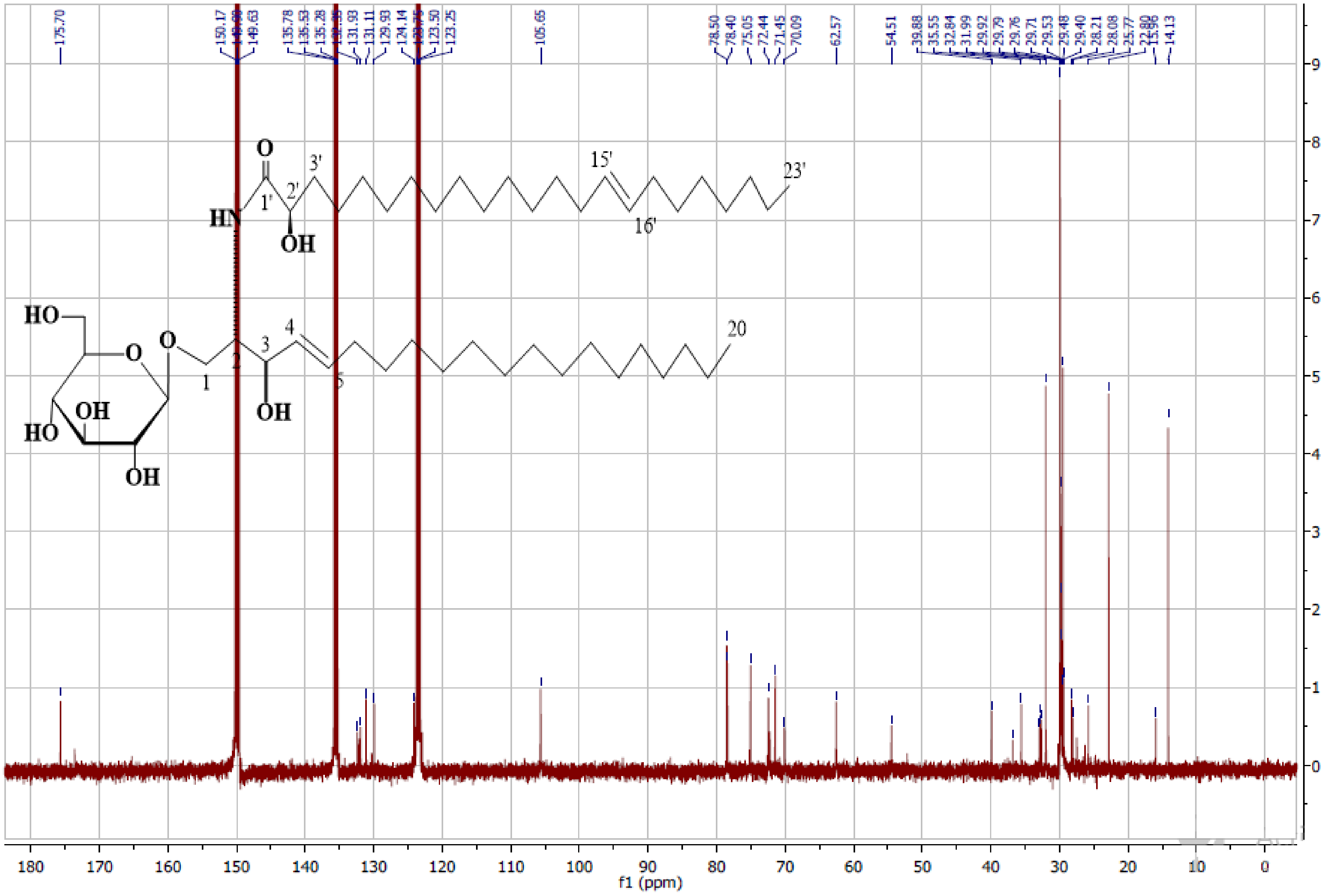

Figure S21. ^{13}C NMR spectrum of compound **2** (in C_5D_5N, 100 MHz).

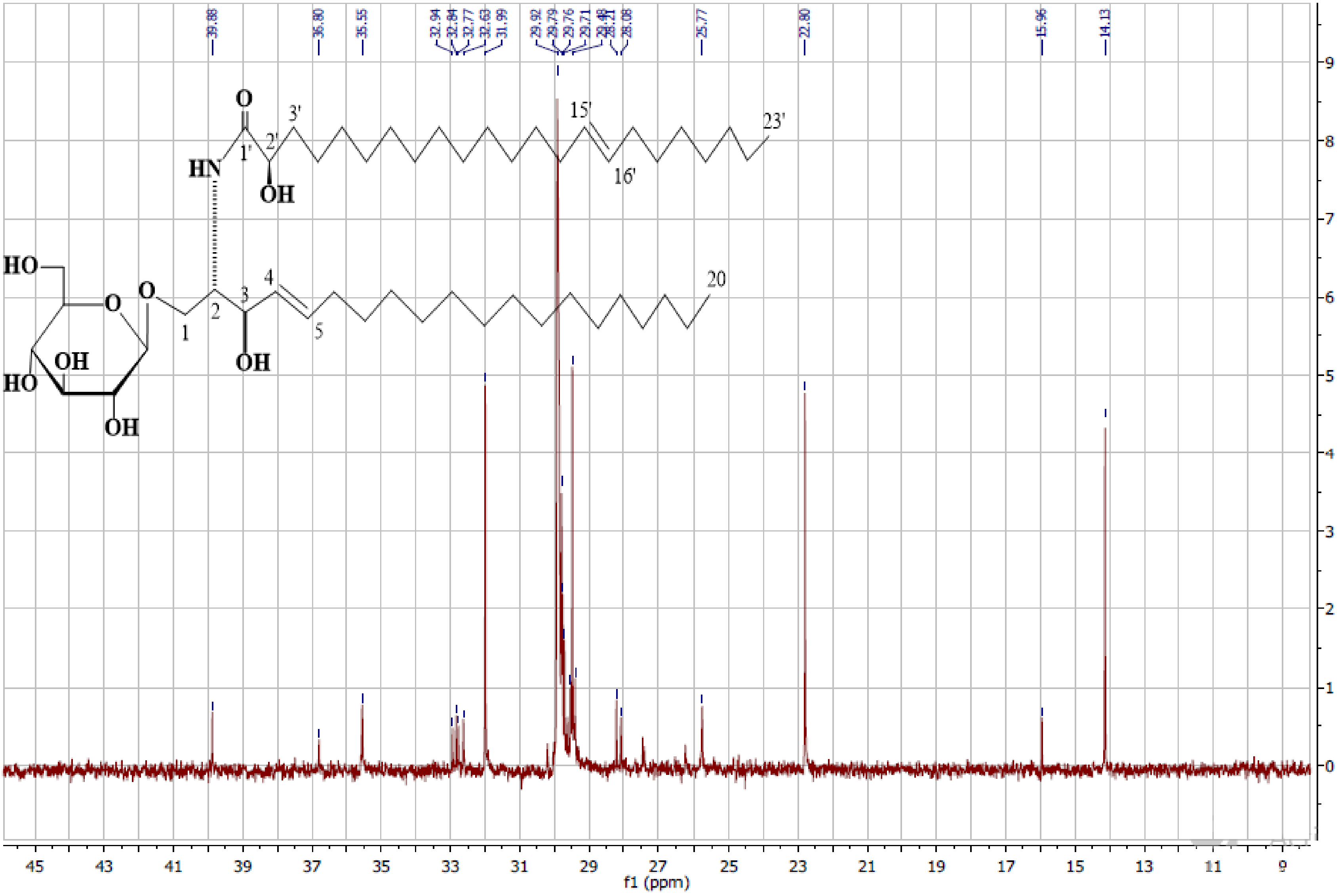

Figure S22. Partial expansion of the ^{13}C NMR spectrum of compound **2** (in C_5D_5N, 100 MHz).

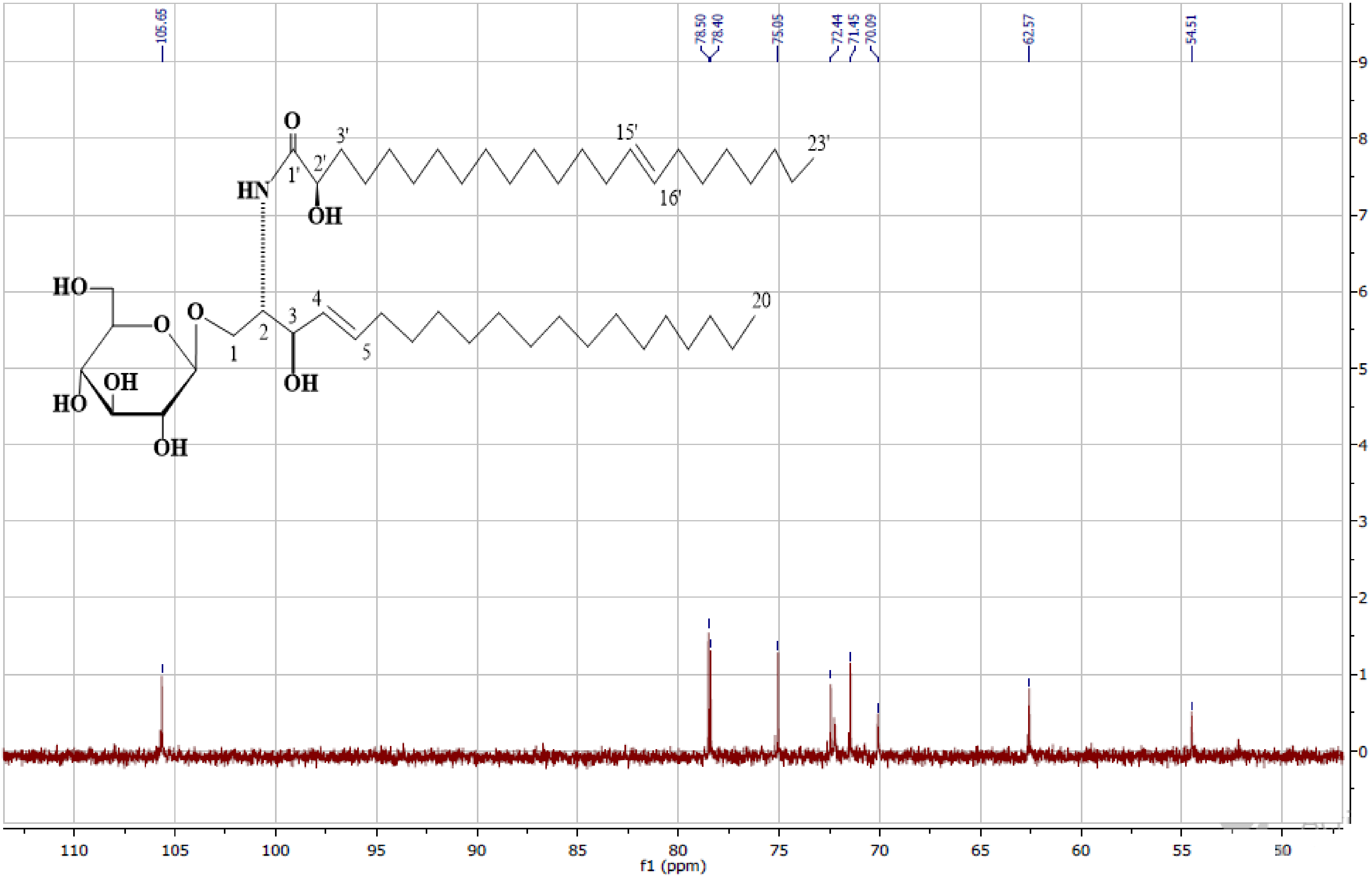

Figure S23. Partial expansion of the ^{13}C NMR spectrum of compound **2** (in C_5D_5N, 100 MHz).

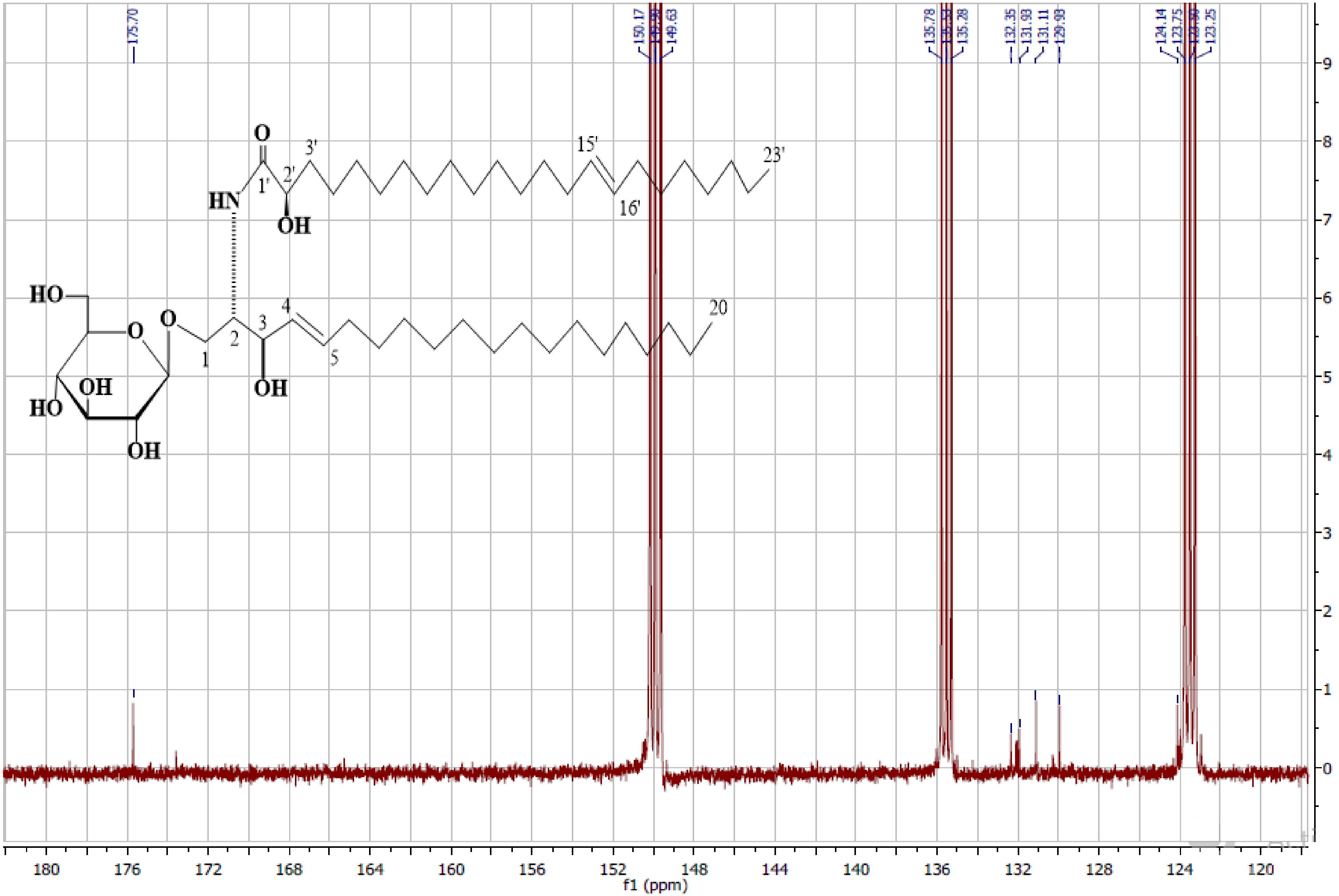

Figure S24. Partial expansion of the ^{13}C NMR spectrum of compound **2** (in C_5D_5N, 100 MHz).

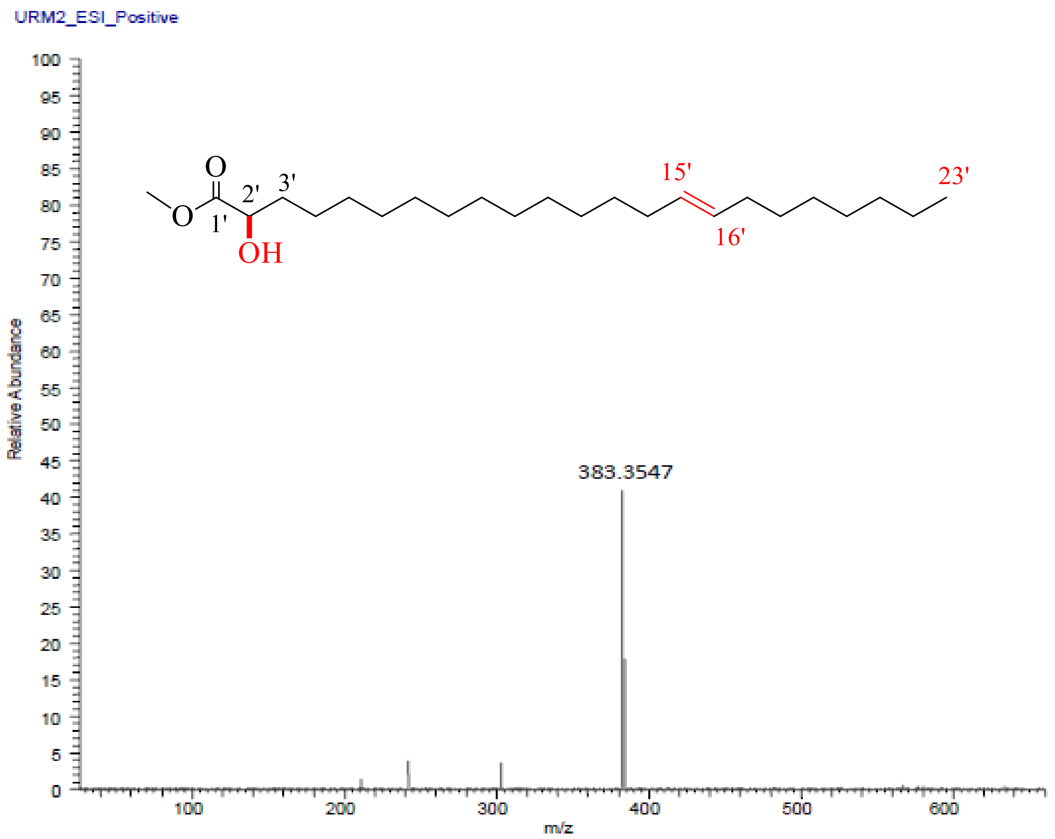

Figure S25. LC-HRESIMS for α-hydroxy fatty acid methyl ester after hydrolysis of compound **2**

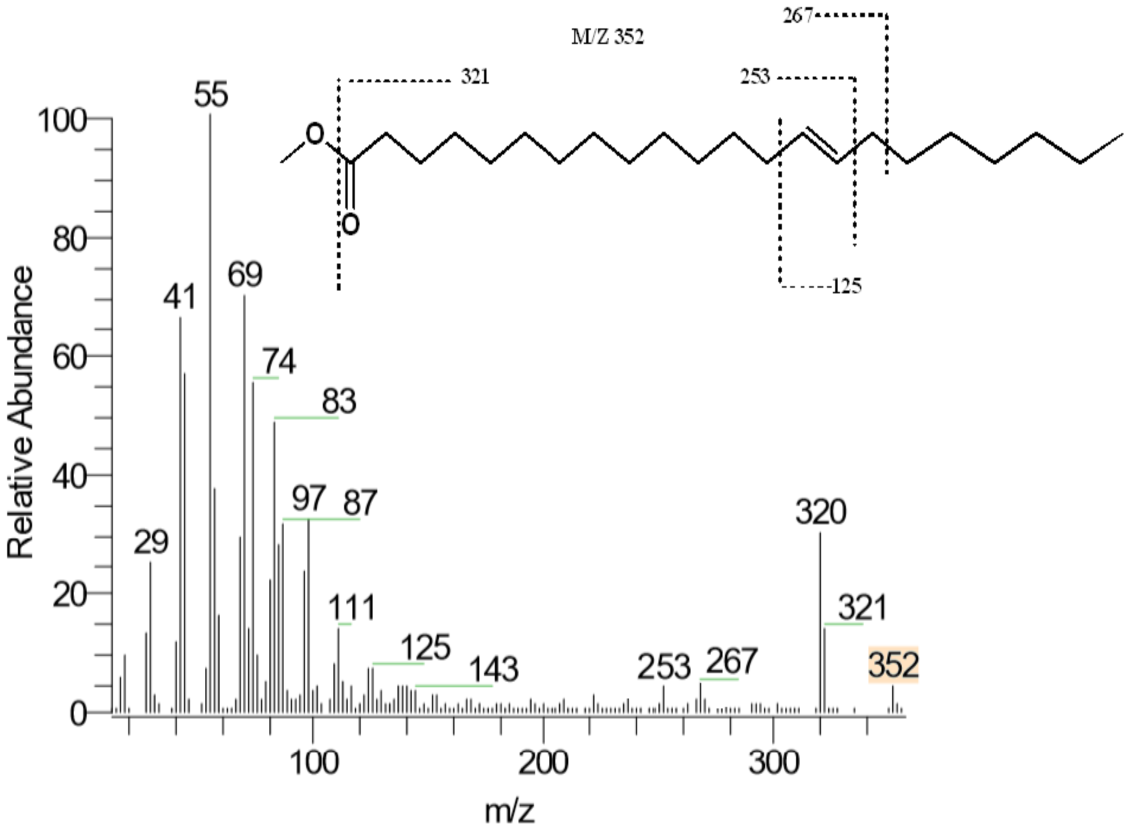

Figure S26. GC-MS analysis of fatty acids methyl esters carried out after oxidation of α-hydroxy fatty acid methyl ester (Compound 2)

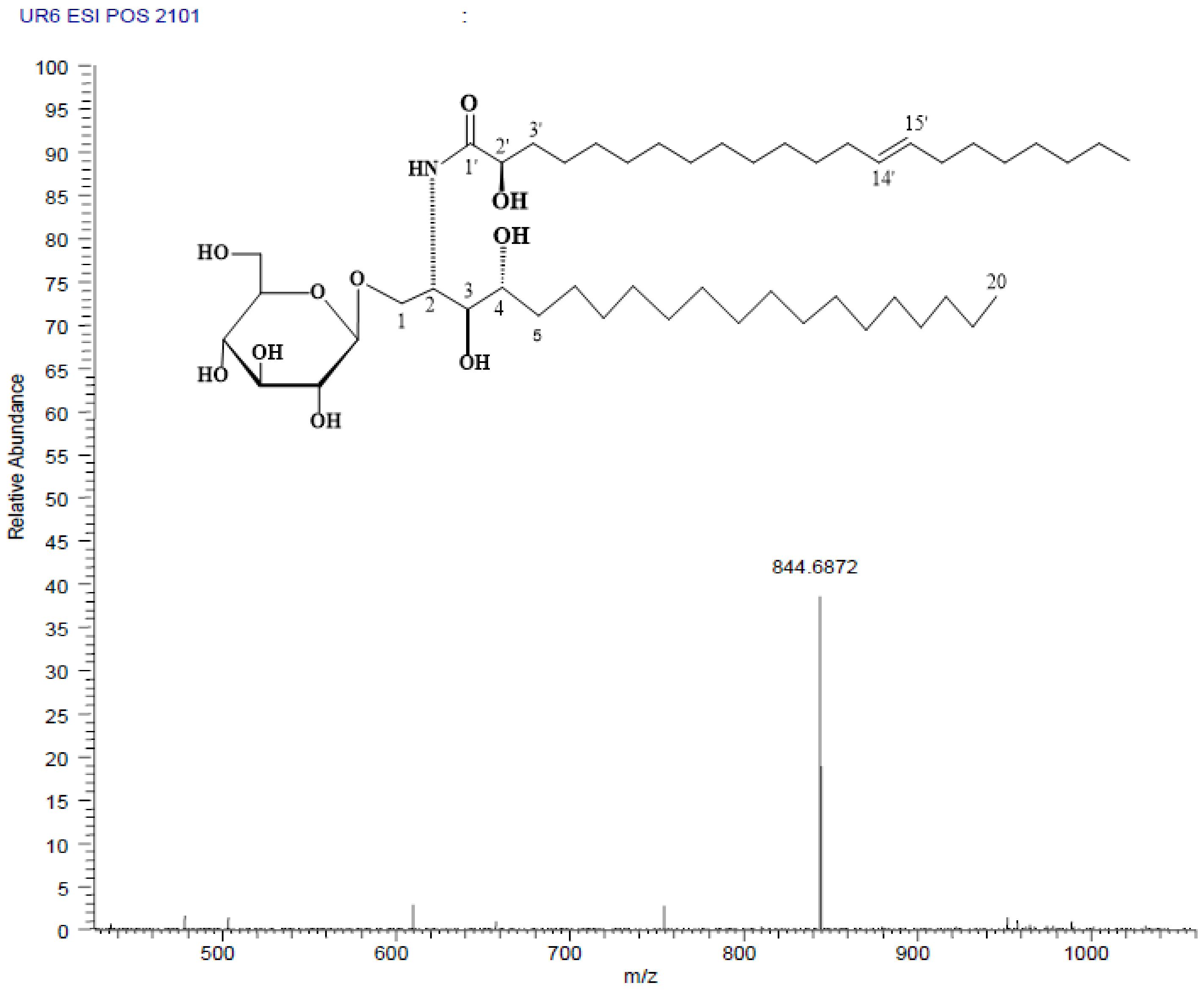

Figure S27. LC-HRESIMS of Compound **3** (M+H)+.

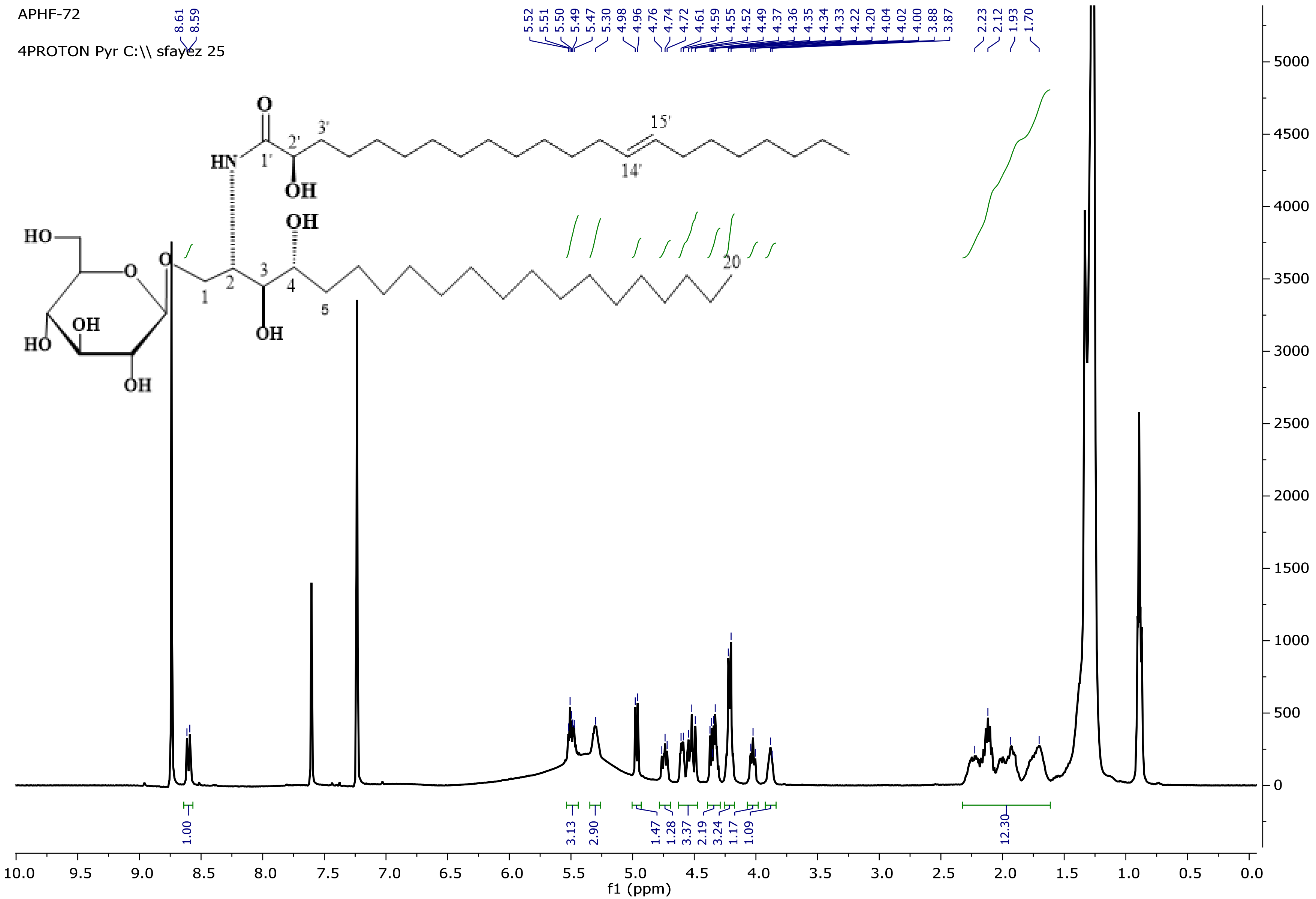

Figure S28. ^{1}H NMR spectrum of compound **3** (in C_5D_5N, 400 MHz).

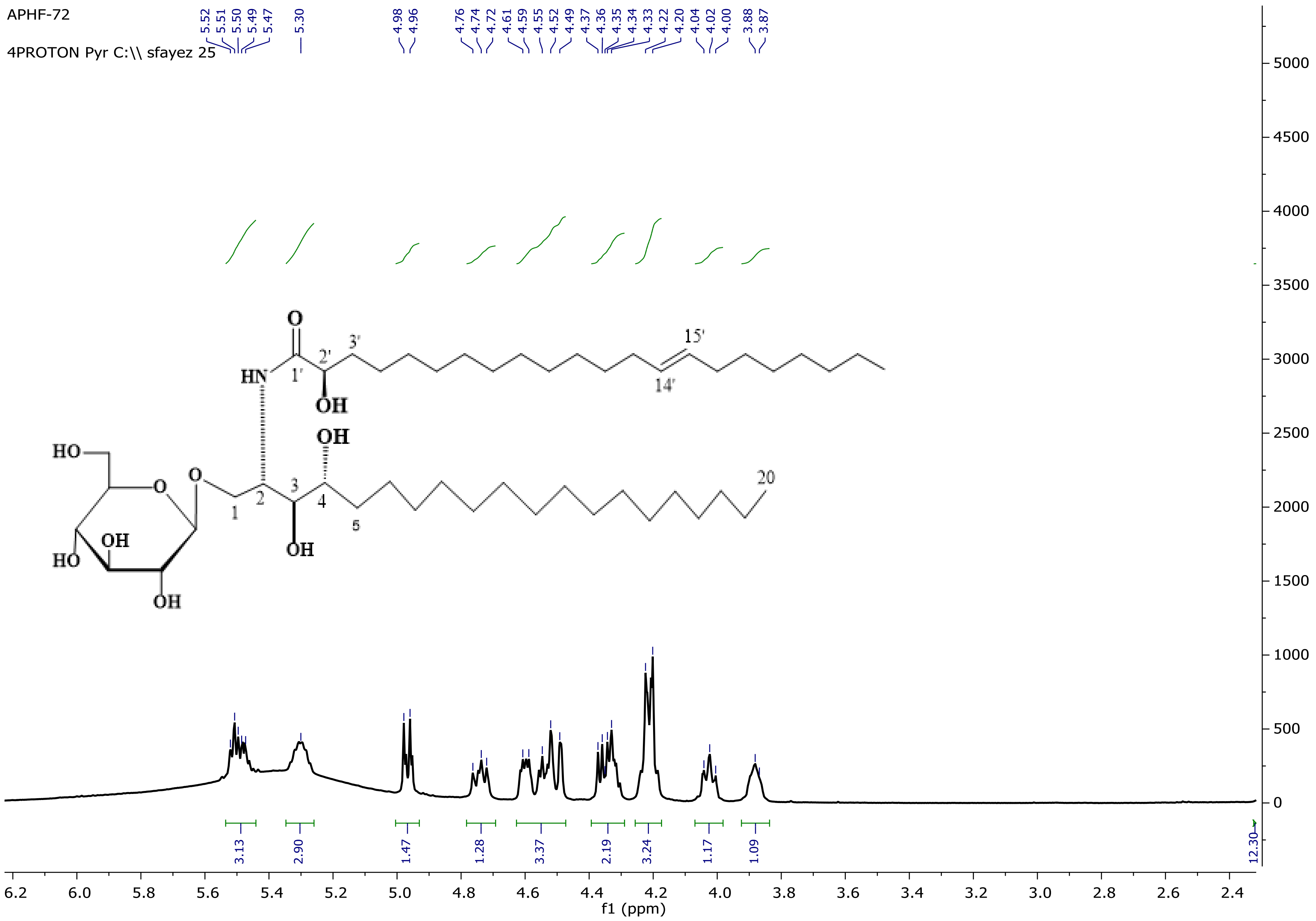

Figure S29. Partial expansion of the ^{1}H NMR spectrum of compound **3** (in C_5D_5N, 400 MHz).

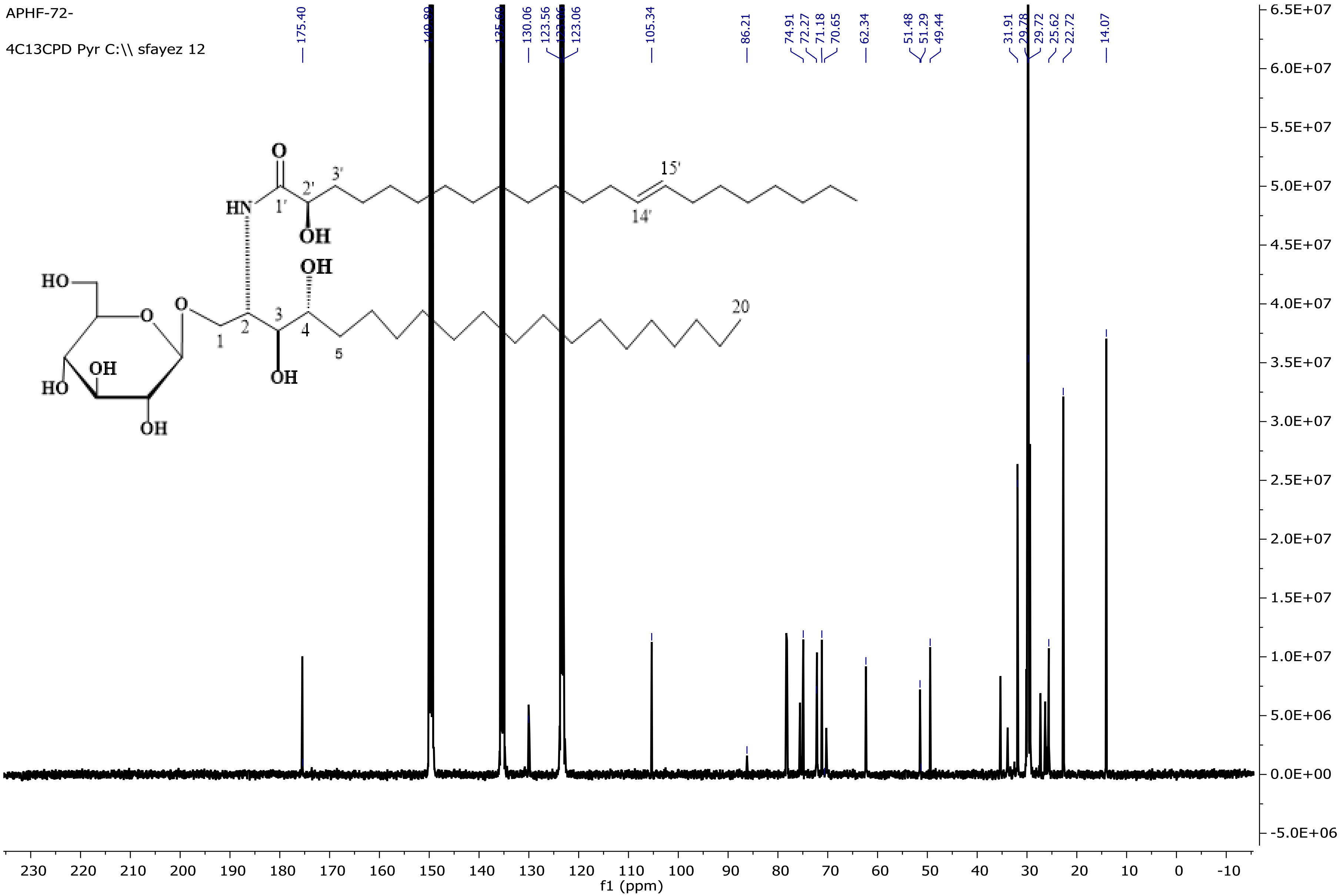

Figure S30. ^{13}C NMR spectrum of compound **3** (in C_5D_5N, 100 MHz).

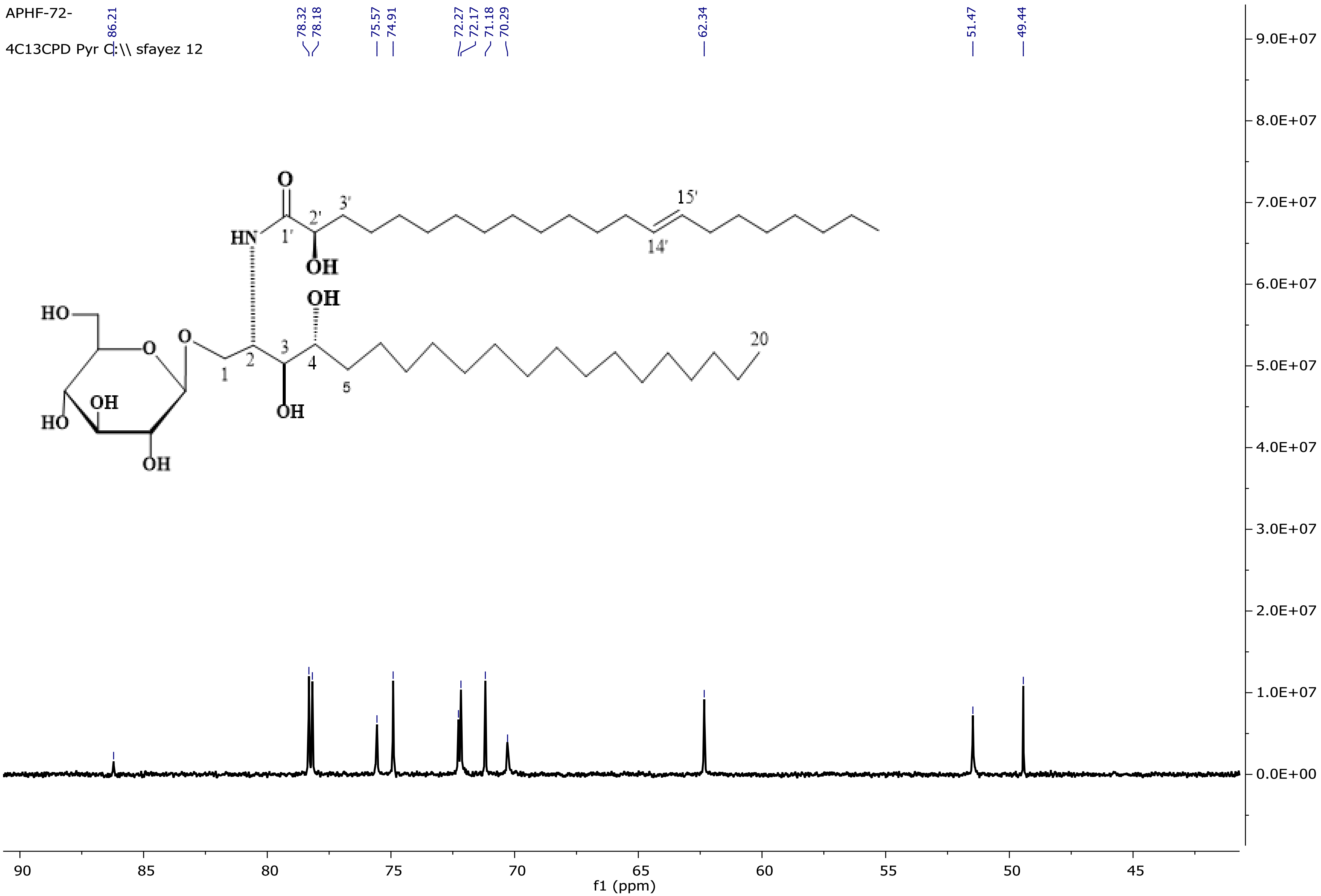

Figure S31. Partial expansion of the ^{13}C NMR spectrum of compound **3** (in C_5D_5N, 100 MHz).

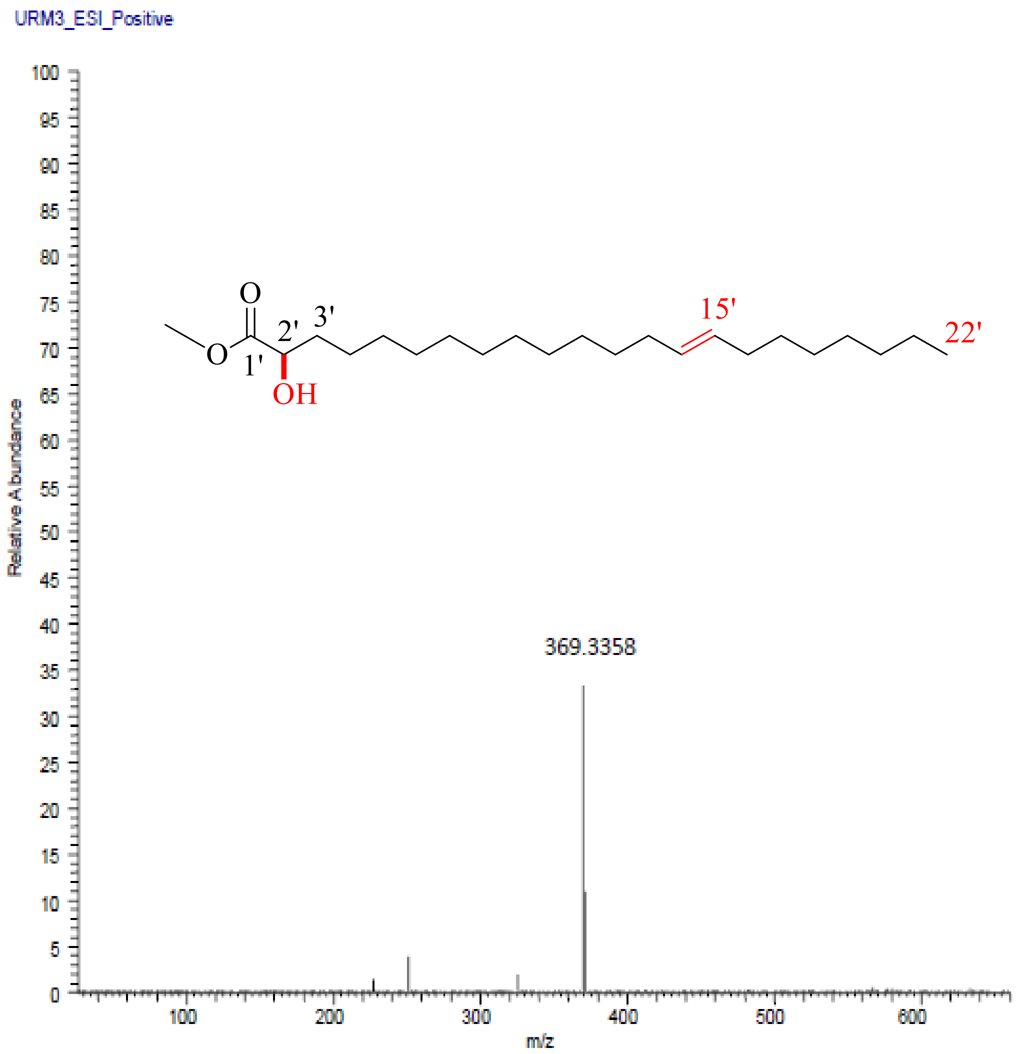

Figure S32. LC-HRESIMS for α-hydroxy fatty acid methyl ester after hydrolysis of compound **3**

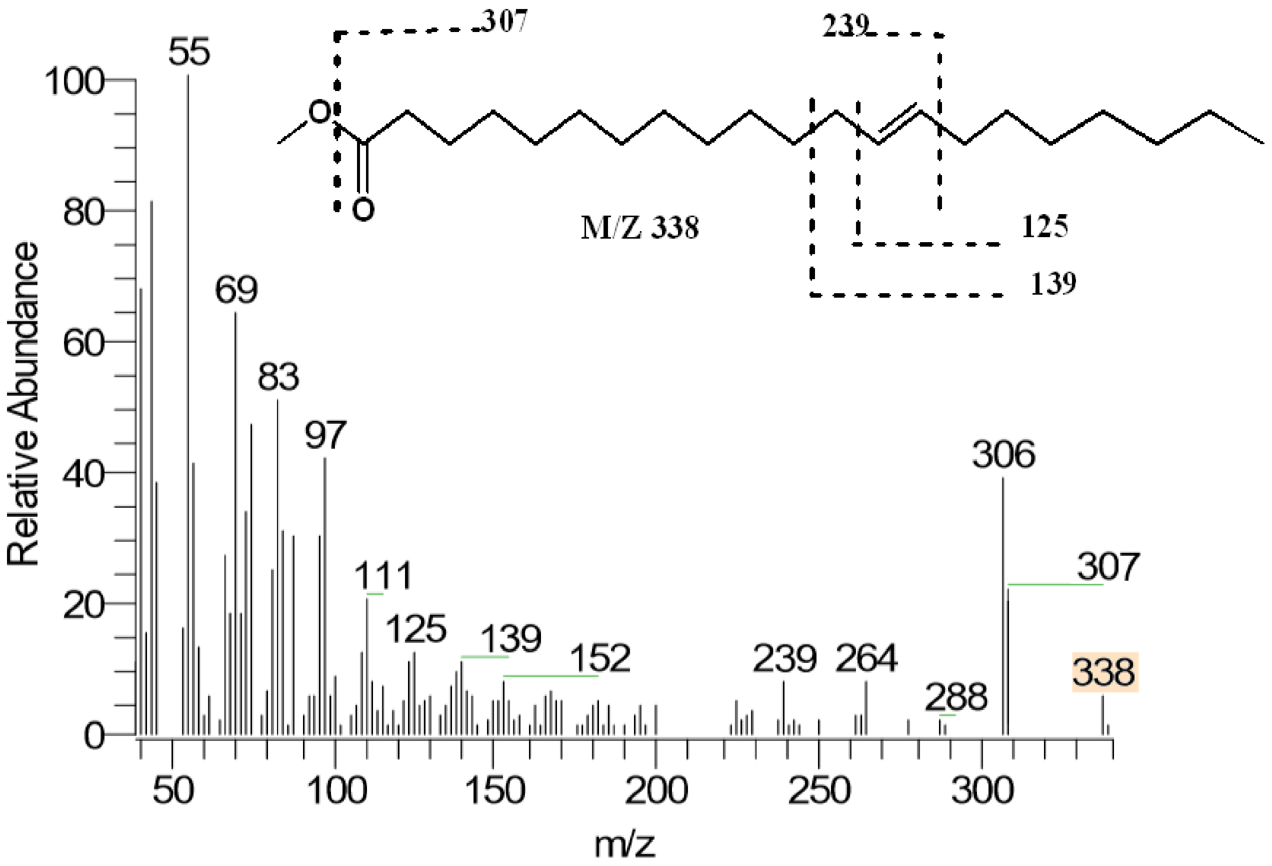

Figure S33. GC-MS analysis of fatty acids methyl esters carried out after oxidation of α-hydroxy fatty acid methyl ester (Compound 3)

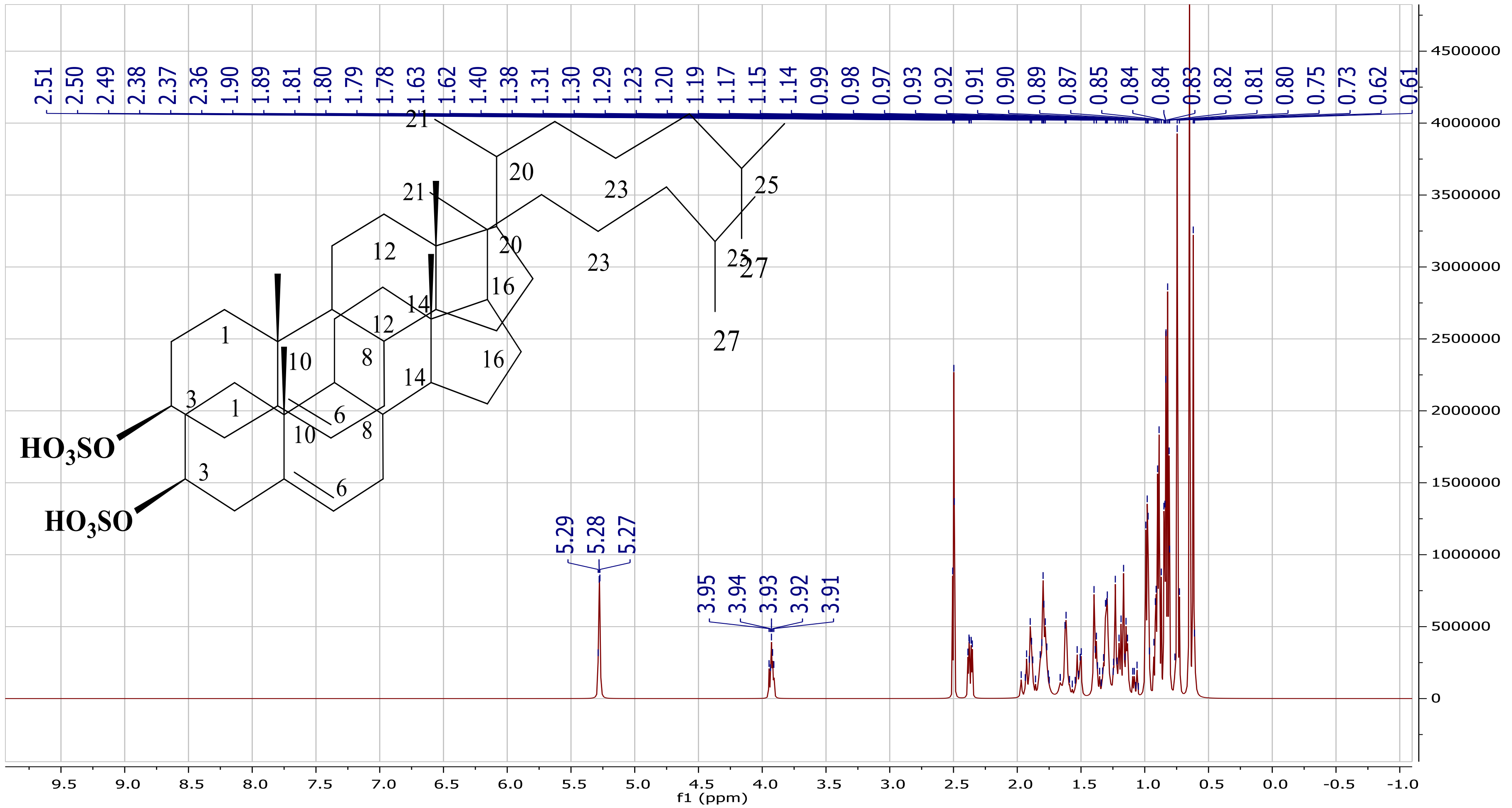

Figure S34. ^{1}H NMR spectrum of compound **4** (in DMSO, 400 MHz)

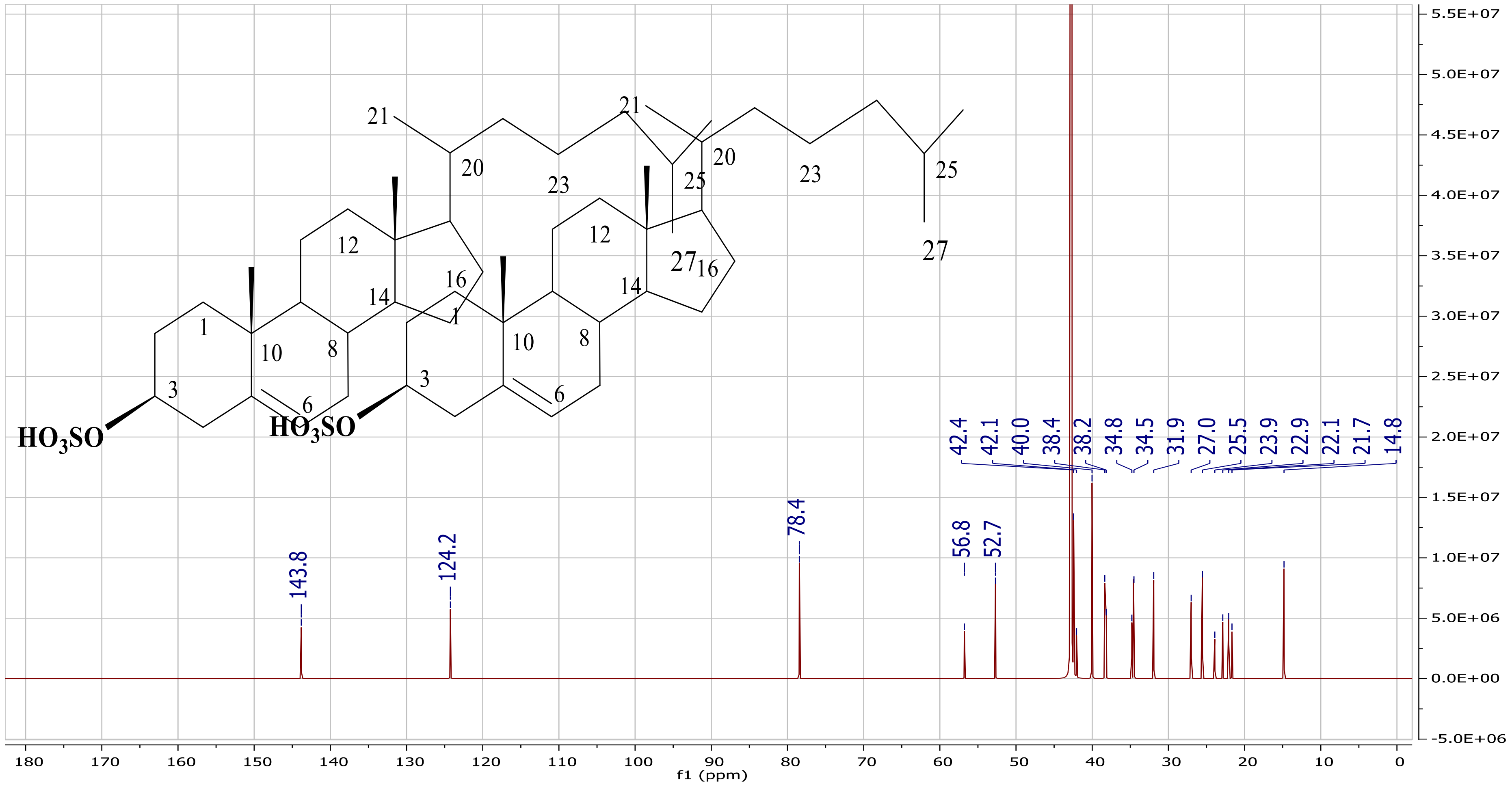

Figure S35. ^{13}C NMR spectrum of compound **4** (in DMSO, 400 MHz)

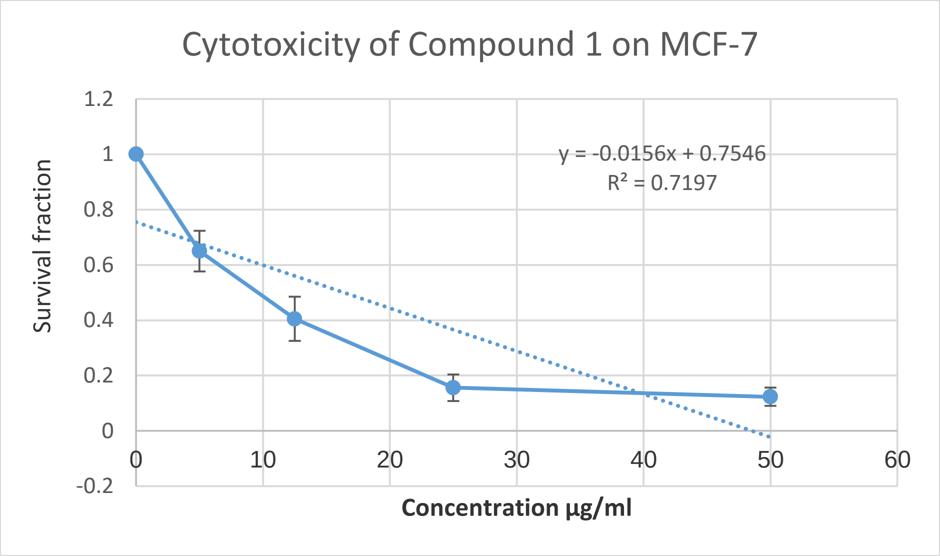

Figure S36. Cytotoxicity of compound **1** on MCF-7

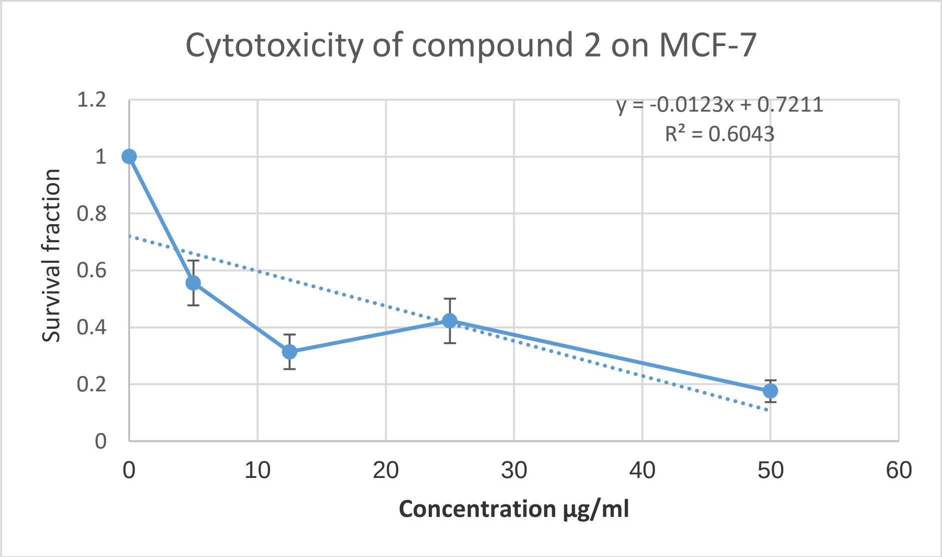

Figure S37. Cytotoxicity of compound **2** on MCF-7

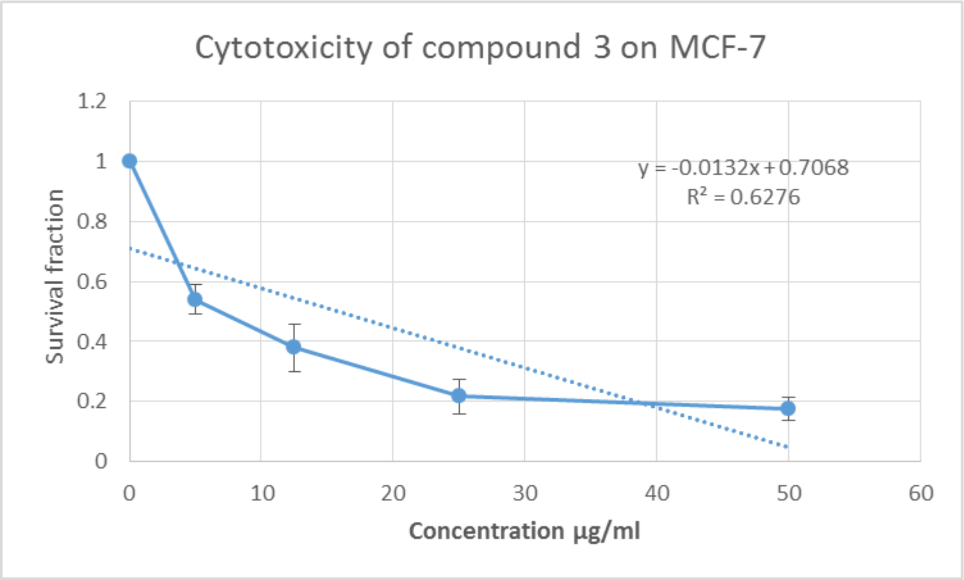

Figure S38. Cytotoxicity of compound **3** on MCF-7

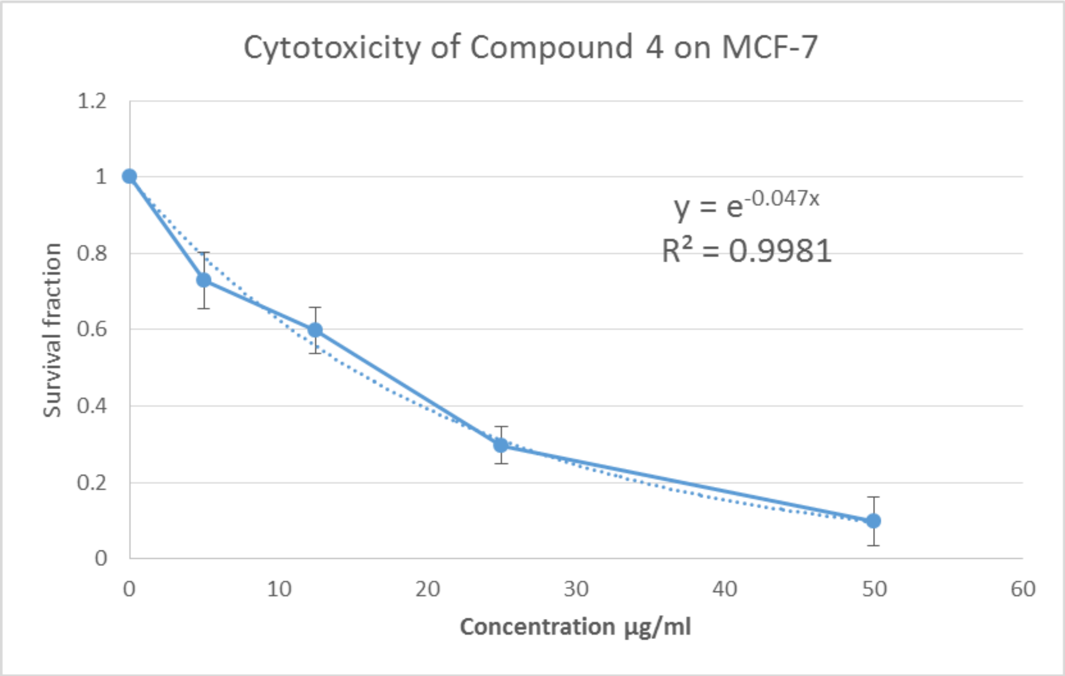

Figure S39. Cytotoxicity of compound **4** on MCF-7

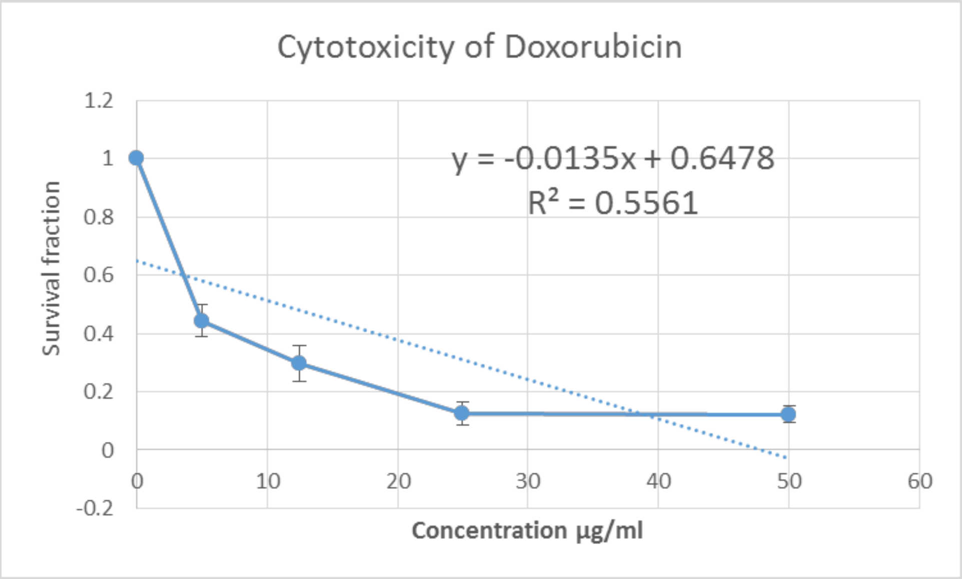

Figure S40. Cytotoxicity of Doxorubicin on MCF-7
